# The Grand Canonical General-Purpose Reactivity Indicator: A Conceptual DFT Approach to Predict Molecular Reactivity and Experimental Electrophilicity and Nucleophilicity Scales

**DOI:** 10.1021/acs.jctc.5c00849

**Published:** 2025-10-02

**Authors:** Yoshio Barrera, Tomás Rocha-Rinza, Florian F. Mulks, Paul W. Ayers, James S. M. Anderson

**Affiliations:** † Instituto de Química, 7180 Universidad Nacional Autónoma de México, Circuito Exterior, Ciudad Universitaria, Delegación Coyoacán, Ciudad de México C.P. 04510, México; ‡ Institute of Organic Chemistry, 9165 RWTH Aachen University, Landoltweg 1, Aachen 52074, Germany; § Department of Chemistry and Chemical Biology, McMaster University, Hamilton, Ontario L8S 4M1, Canada

## Abstract

The Grand Canonical General-Purpose Reactivity Indicator (GC-GPRI) is introduced as a tool for predicting reactivity and for discerning the relative electrophilicity and nucleophilicity of electrophiles and nucleophiles, respectively. The GC-GPRI is derived within the zero-temperature grand canonical ensemble conceptual density-functional theory (CDFT) framework using a perturbative approach. In this model, the electrophile–nucleophile interaction energy is modeled by perturbations in the chemical and external potentials of the isolated species. The GC-GPRI accurately identifies the most reactive hard and soft atoms in complex molecules with multiple reactive sites. This model also reproduces experimental electrophilicity and nucleophilicity scales for 21 electrophiles and 20 nucleophiles, with *R* ^2^ correlations of 0.98 and 0.94, respectively.

## Graphical abstract

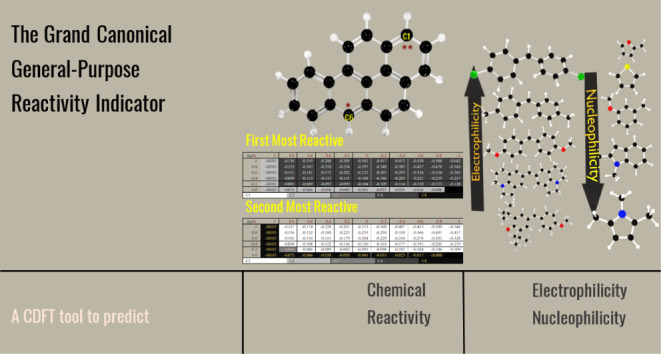

## 1 Introduction

Electrophilicity and nucleophilicity are fundamental molecular properties that define the ability of a chemical species to accept or donate electrons during chemical reactions, respectively.^1–3^ These characteristics play a central role in determining regioselectivity and determining overall reaction outcomes.^4,5^ In this context, the quantification and prediction of electrophilic and nucleophilic behavior at both global and local levels are essential for developing theoretical models of chemical reactivity,^6–11^ and for identifying efficient and selective synthetic routes.^12–14^

Experimentally, electrophilicity and nucleophilicity have been characterized using parameters such as rate constants,^2,15–19^ as well as by analyzing how these rate constants vary with different substituents.^4,20,21^ A major contribution in this field is the work of Mayr and coworkers, who developed comprehensive scales to quantitatively rank the relative electrophilicity and nucleophilicity of a wide range of chemical species.^19,22–25^ These scales have become invaluable tools for comparing molecular reactivity and have supported the development of machine learning models for predicting selectivity.^26,27^ Despite its recent growth, many models continue to rely on data derived from established frameworks such as conceptual DFT (CDFT) for training.^28–30^

Descriptors derived from CDFT have played a leading role in understanding chemical reactivity at both global and local levels.^31–34^ CDFT provides a rigorous and physically grounded framework for relating the reactivity of molecules with their electronic structures.^31^ Among the global descriptors, based on changes in electronic energy with respect to the number of electrons are electronegativity,^35,36^ hardness,^37–41^ softness,^42–44^ chemical potential,^42,45^ and electrophilicity.^9,27,28,46^ These aim to capture the overall reactivity of two electronic systems, for example by predicting which of the two species is more likely to donate or to accept electrons. Local descriptors such as atomic charges,^47,48^ Fukui functions,^49^ electrostatic potentials,^50,51^ frontier molecular orbitals,^52,53^ general-purpose reactivity indicators,^54–63^ and dual descriptors,^64,65^ among others,^7,42,66–70^ are widely employed to identify the most reactive sites within molecules. Despite the extensive development of these tools, relatively few models are capable of simultaneously capturing both global and local reactivity.^47,48^ Moreover, these approaches are grounded in either charge transfer or frontier orbital interactions, which makes them incapable of finely distinguishing multiple reactive sites within ambident molecules. To address these limitations we introduce the Grand Canonical General-Purpose Reactivity Indicator (GC-GPRI), a novel two-parameter descriptor grounded in CDFT.^31,32^ Building on the limitations of existing local and global descriptors, the GC-GPRI offers a unified framework capable of capturing both behaviors. This model is derived from the interaction energy between an electrophile and a nucleophile using a perturbative treatment of the isolated species that account for variations in chemical potential (μ) and external potential (ν(r)) within the zero-temperature grand canonical ensemble.^46,71,72^ By integrating electron transfer, and electrostatic contributions the GC-GPRI enables the elucidation of chemical reactivity and it is able to reproduce relative experimental electrophilicity and nucleophilicity scales.

This paper is structured into five sections. In Section 2 the derivation of the GC-GPRI model is detailed, while in Section 3 the theoretical methodology is described. In Section 4, we present the results from applying the GC-GPRI model in elucidating the reactivity of complex molecules and its correlation with experimental relative electrophilicity and nucleophilicity. Section 5 outlines the key conclusions of this study.

## 2 Derivation

In the spirit of the original GPRI,^54^ we expanded the interaction energy between a target molecule and an attacking reagent using a perturbative approach^46^ within the zero-temperature grand-canonical (GC) ensemble description in CDFT. This formulation is based on the grand potential, Ω,^31,32,73^

1 Ω=E−Nμ

where *E* is the electronic energy, *N* is the number of electrons, and μ is the chemical potential of the target molecule.^45^ The potential energy surface, *U*, is described by the Born–Oppenheimer approximation^54,74^ as the sum of the electronic energy and the nuclear–nuclear repulsion energy, *V_nn_*,

2 $$U=E+V_{nn}$$

The substitution of eq 1 into eq 2 yields,

3 $$U=V_{nn}+N\mu +\Omega$$

The changes in the interaction potential energy surface (Δ*U*) are expressed as a Taylor series expansion in terms of the chemical potential, *μ*, and the external potential, *ν*(***r***), which are the natural variables of the grand potential, Ω = Ω[*μ*,*ν*(***r***)].^72,75–77^ Although the series theoretically contains an infinite number of terms,^78^ it is typically truncated to retain only the first derivatives with respect to *μ* and *ν*(***r***), along with the mixed second derivative that accounts for their combined variations. These contributions are typically much larger than the other second derivatives and higher-order corrections.^54^ This procedure leads to the following equation,

4 $$\Delta U=\int \frac{\delta V_{nn}\left[\nu \right]}{\delta \nu \left(r\right)}\Delta \nu \left(r\right)dr+N\Delta \mu +{\left(\frac{\partial \Omega }{\partial \mu }\right)}_{\nu \left(r\right)}\Delta \mu +\int {\left(\frac{\delta \Omega }{\delta \nu \left(r\right)}\right)}_{\mu }\Delta \nu \left(r\right)dr+\left\{\int {[\frac{\partial }{\partial \mu }{\left(\frac{\delta \Omega }{\delta \nu \left(r\right)}\right)}_{\mu }]}_{\nu \left(r\right)}\Delta \nu \left(r\right)dr\right\}\Delta \mu +...+$$

which describes the change in the interaction potential energy surface between the target molecule and the modeled attacking species. Now, we describe every term in the right-hand side of eq 4 according to the grand canonical ensemble in CDFT.^31^ The first term represents the change in nuclear–repulsion energy due to variations in *ν*(***r***), which is detailed in Appendix A of the work of Anderson et al.,^54^

5 $$\frac{\delta V_{nn}\left[\nu \right]}{\delta \nu \left(r\right)}=-\rho_{nuc}\left(r\right)=-\underset{\alpha }{\sum }Z_{\alpha }\delta \left(r-R_{\alpha }\right)$$

where *ρ_nuc_* (***r***) represents the nuclear charge density, δ is the Dirac delta function, and *Z*_α_ and ***R***_α_ denote the charge and position of each nucleus *α* of the target molecule (i.e., a nucleophile), respectively. The second term corresponds to the change in the chemical potential at a constant number of electrons from eq 3. The third term includes the partial derivative of the grand potential with respect to the chemical potential, with the external potential fixed (from eq 1). This quantity equals the negative of the number of electrons, ${\left(\frac{\partial \Omega }{\partial \mu }\right)}_{\nu \left(r\right)}=-N$ . Therefore, this term and the second cancel each other. The fourth term involves the functional derivative of the grand potential with respect to the external potential at a constant number of electrons or the electron density (*ρ*(***r***)).^31^ The fifth term is the variation of the grand potential with respect to both the external and chemical potentials. This contribution can be rewritten as the partial derivative of the electron density with respect to the chemical potential at constant external potential, and, by using the chain rule, we obtain,

[(∂∂μ(δΩvδν(r))μ)]ν(r)=(∂ρ(r)∂μ)ν(r)=(∂ρ(r)∂N)ν(r)(∂N∂μ)ν(r)=f(r)S=s(r) 6

where $f\left(r\right)={\left(\frac{\partial \rho \left(r\right)}{\partial N}\right)}_{\nu \left(r\right)}$ is the Fukui function,^79,80^ $S={\left(\frac{\partial N}{\partial \mu }\right)}_{\nu \left(r\right)}$ is the global softness,^81^ and their product yields the local softness, *s*(***r***). Taking into account these considerations into eq 4 leads to an approximate model for the interaction between the two reactants,

7 $$\Delta U=\int \underset{\alpha }{\sum }-Z_{\alpha }\delta \left(r-R_{\alpha }\right)\Delta \nu \left(r\right)dr+\int \rho \left(r\right)\Delta \nu \left(r\right)dr+\left\{\int s\left(r\right)\Delta \nu \left(r\right)dr\right\}\Delta \mu$$

There are two distinct scenarios described in eq 7. The first one occurs when the molecule of interest acts as a nucleophile (*Nu*), and the attacking species is an electrophile (*Ele*). In this case, the energy change is given by,

8 ΔUNu=−∫(∑α∈NuZαδ(r−Rα)−ρNu(r)−ΔμsNu−(r))ΔνEle(r)dr

where $s_{Nu}^{-}\left(r\right)$ represents the local softness of the nucleophile, and the change in the external potential due to the electrons and nuclei of the attacking electrophile, Δ*ν_Ele_* (***r***). Although an exact expression for Δ*ν_Ele_* (***r***) is available,^82^ it is unwieldy. However, when the reagents are well-separated,^46,78^ the following expression can be used,

9 $$\Delta \nu_{Ele}\left(r_{p}\right)=-\int \frac{\underset{\beta \in Ele}{\sum }Z_{\beta }\delta \left(r-R_{\beta }\right)-\left[\rho_{Ele}\left(r\right)+\left(-\Delta \mu \right)s_{Ele}^{+}\left(r\right)\right]}{\left|r-r_{p}\right|}dr=-\Phi_{Ele}\left(r_{p}\right)-\Delta \mu \int \frac{s_{Ele}^{+}\left(r\right)}{\left|r-r_{p}\right|}dr$$

Here Δ*ν_Ele_* (***r***) is evaluated at the reactive site ***r**_p_*. This expression is approximated by the negative electrostatic potential (Φ*_Ele_* (***r**_p_*)), including a correlation term to empirically account for electron transfer effects not captured by the electrostatic potential alone.^54^ This correction term (the second term on the right-hand side) is expressed as the product of the change in the chemical potential (Δ*μ*) and an integral involving the softness potential, which is closely related to the Fukui function and thus captures electron transfer behavior.

Analogously to eq 8, the change in the interaction energy for an electrophile undergoing a nucleophilic attack is expressed as,

10 $$\Delta U_{Ele}=-\int \left(\underset{\beta \in Ele}{\sum }Z_{\beta }\delta \left(r-R_{\beta }\right)-\rho_{Ele}\left(r\right)+\Delta \mu s_{Ele}^{+}\left(r\right)\right)\Delta \nu_{Nu}\left(r\right)\;dr$$

where *Z*_*β*_ and ***R***_*β*_ represent the nuclear charge and position of each nucleus *β* in the electrophile, respectively. The term $s_{Ele}^{+}\left(r\right)$ corresponds to the local softness appropriate to describe nucleophilic attacks, and Δν*_Nu_* (***r***) denotes the change in the external potential of attacking nucleophile. When evaluated at the reactive site, ***r**_p_*, Δν*_Nu_* is approximated as,^54^

11 $$\Delta \nu_{Nu}\left(r_{p}\right)=-\int \frac{\underset{\alpha \in Nu}{\sum }Z_{\alpha }\delta \left(r-R_{\alpha }\right)-\left[\rho_{Nu}\left(r\right)+\Delta \mu s_{Nu}^{-}\left(r\right)\right]}{\left|r-r_{p}\right|}dr=-\Phi_{Nu}\left(r_{p}\right)+\Delta \mu \int \frac{s_{Nu}^{-}\left(r\right)}{\left|r-r_{p}\right|}dr$$

The sign change for the terms $\Delta \mu s_{Nu}^{-}\left(r\right)$ and $\Delta \mu s_{Ele}^{+}\left(r\right)$ in eqs 8 and 10, respectively (and correspondingly in eqs 9 and 11) reflects chemical potential equalization during electrophile-nucleophile interactions.^31,45,83^

To model the interaction energy (Δ*U_int_*) between a nucleophile and an electrophile, we considered the scenarios of an electrophile attacking a nucleophile and vice versa. Therefore, eqs 9 and 11 are substituted into eqs 8 and 10, respectively, and the resulting Δ*U_int_* is given by Δ*U_int_* = Δ*U_Ele_* + Δ*U_Nu_*, with a correction to avoid double counting of the identical electrostatic interaction,

12 $$\Delta U_{int}=\int \left(\underset{\alpha \epsilon Nu}{\sum }Z_{\alpha }\delta \left(r-R_{\alpha }\right)-\rho_{Nu}\left(r\right)\right)\Phi_{Ele}\left(r\right)dr+\Delta \mu \int \left(s_{Ele}^{+}\left(r\right)\Phi_{Nu}\left(r\right)-s_{Nu}^{-}\left(r\right)\Phi_{Ele}\left(r\right)\right)dr-{\left(\Delta \mu \right)}^{2}\int \int \frac{s_{Nu}^{-}\left(r\right)s_{Ele}^{+}\left(r^{\prime }\right)}{\left|r-r^{\prime }\right|}drdr^{\prime }$$

The first term on the right-hand side of eq 12 represents the electrostatic interaction between the nucleophile and electrophile. The subsequent term, proportional to Δ*μ*, describes the interplay between electrostatic and electron transfer behaviors. The third term, proportional to Δ*μ* ^2^, reflects electron transfer behavior^84^ and it is related to frontier molecular orbital theory.^52^

Reactants minimize the interaction energy as they approach, with stronger attraction leading to more negative interaction energies and greater reactivity. Thus, a more negative interaction energy indicates a higher reactivity. The GPRI models are most reliable for reactions which involve early transition states or kinetic control.^85,86^

Focusing on monodentate attacking molecules, we can approximate their reactivity by the properties of their active site. The specific identity of the attacking species is not essential.^79,82^ As such, the attacking electrophile can be treated as a model perturbation.^54^ Accordingly, in eq 13, the electrostatic potential of the electrophile (Φ*_Ele_* (***r***)) is approximated as a point charge $\left(q_{Ele}^{\left(0\right)}\right)$ located at the reactive site,

13 $$\Phi_{Ele}\left(r\right)\approx \frac{q_{Ele}^{\left(0\right)}}{\left|r-R_{Ele}\right|}$$

The point charge approximation simplifies the electrostatic treatment and yields a more workable equation that is compatible with the condensed softness formulation (see eq 22). Despite its simplicity, this approximation is enhanced by the subsequent inclusion of the correction parameter ${\mathrm{\kappa ̃}}_{Ele}$ , which accounts for electron transfer contributions.^54^

The local softness of the electrophile is replaced with its condensed version,

$$s_{Ele}^{+}\left(r\right)\approx s_{Ele}^{\left(+\right)}\delta \left(r-R_{Ele}\right)$$ 14

while the so-called, softness potential of the nucleophile, $\nu_{Nu}^{s^{-}}\left(r\right),$ is approximated as,^43,87^

15 $$\nu_{Nu}^{s^{-}}\left(r\right)\approx \frac{s_{Nu}^{-}}{\left|r-R_{Nu}\right|}$$

Applying these approximations to eq 12 yields the electrophile–nucleophile interaction energy for the electrophilic attack to the reactive site at point ***r**_p_* of the target nucleophile,

16 $$\Delta U_{int}\approx \left(q_{Ele}^{\left(0\right)}+\Delta \mu s_{Ele}^{\left(+\right)}\right)\Phi_{Nu}\left(r_{p}\right)-\Delta \mu \left(q_{Ele}^{\left(0\right)}+\Delta \mu s_{Ele}^{\left(+\right)}\right)\nu_{Nu}^{s^{-}}\left(r_{p}\right)$$

Here, the key parameter for the attacking electrophiles, ${\mathrm{\kappa ̃}}_{Ele}$ , is defined as,^54^

17 $${\mathrm{\kappa ̃}}_{Ele}\equiv q_{Ele}^{\left(0\right)}+\Delta \mu s_{ele}^{\left(+\right)}$$

${\mathrm{\kappa ̃}}_{Ele}$ modulates the electrostatic and electron-transfer contributions in the interaction energy model. The GC-GPRI for nucleophiles susceptible to electrophilic attack, denoted by the captial theta character, ${\mathrm{\Theta ̃}}_{\Delta \mu \leq 0}^{{\mathrm{\kappa ̃}}_{Nu}}\left(r_{p}\right)$ , is given by,

18 $${\mathrm{\Theta ̃}}_{\Delta \mu \leq 0}^{{\mathrm{\kappa ̃}}_{Nu}}\left(r_{p}\right)={\mathrm{\kappa ̃}}_{Ele}\Phi_{Nu}\left(r_{p}\right)-{\mathrm{\kappa ̃}}_{Ele}\Delta \mu \nu_{Nu}^{s^{-}}\left(r_{p}\right)$$

In eq 18, the attacking molecule is treated as a point–charge with corrections, defined in eq 17, which overly simplifies the interaction.^46,54^ To circumvent this problem, Anderson et al.^54^ introduced corrections for the electron density (*ϵ*_*ρ*_) and electron transfer behavior (*ϵ*_*s*_) of the attacking electrophile, resulting in the following expression,

19 $${\mathrm{\Theta ̃}}_{\Delta \mu \leq 0}^{{\mathrm{\kappa ̃}}_{Nu}}\left(r_{p}\right)=\left({\mathrm{\kappa ̃}}_{Ele}+\epsilon_{\rho }\right)\Phi_{Nu}\left(r_{p}\right)-\left({\mathrm{\kappa ̃}}_{Ele}+\epsilon_{s}\right)\Delta \mu \nu_{Nu}^{s^{-}}\left(r_{p}\right)$$

The correction terms (*ϵ*_*ρ*_ and *ϵ*_*s*_) are defined on an electronic charge scale detailed in ref 54. As a result of this procedure, *κ* arises as a replacement for *κ̃*_*E*_ *_le_* , *ϵ*_*ρ*_ and *ϵ*_*s*_,

20 $$\Theta_{\Delta \mu \leq 0}^{\kappa }\left(r_{p}\right)=\left(\kappa +1\right)\Phi_{Nu}\left(r_{p}\right)-\Delta \mu \left(\kappa -1\right)\nu_{Nu}^{s^{-}}\left(r_{p}\right)$$

This approximation avoids the direct calculation of these error terms, since *κ* is an arbitrary parameter which includes electron density, and the electron transfer corrections to the electrostatic potential (Φ_*Nu*_(***r***_*p*_)) and to the softness potential $\left(\nu_{Nu}^{s^{-}}\left(r_{p}\right)\right)$ of the target molecule, respectively. The *κ* term modulates the reactivity conditions from charge (or electrostatic) reactions (*κ* ≈ 1) to electron-transfer reactions (*κ* ≈ −1) and for the intermediate between both situations (*κ* ≈ 0). Moreover, *κ* contains information about the attacking electrophile regarding its electrostatic and electron-transfer behavior (as discussed below eq 17).

The electron transfer behavior of the reaction is regulated by the chemical potential equalization between two reactants *A* and *B*,^32,40^ which can be estimated employing a quadratic model for the interaction energy in terms of the change in number of electrons (Δ*N*) and hardness of the corresponding species (*η*),^88^

21 $$\Delta N\approx \frac{\mu_{B}-\mu_{A}}{\eta_{A}+\eta_{B}}$$

In most reactions |Δ*N*|<1, and hardness values are generally below 0.5 hartree. For instance, the helium atom has a hardness of approximately 0.5 hartree,^89^ which is higher than that of most species. This observation indicates that 1 hartree is a reasonable upper limit in the denominator of eq 21. Therefore, Δ*μ* can be approximated with the same bounds as Δ*N*.^90^ However, when both reactants are known, Δ*μ* can be calculated employing eq 21.^91^

Eq 20 enables the identification of the most reactive regions of a molecule using electrostatic potential maps and softness potential surfaces. In this study, however, we focus on quantitative reactivity values by replacing the molecular electrostatic potential, Φ_*Nu*_(***r***_*p*_), with that generated by the ground-state atomic charge of each *α* atom, $q_{Nu,\alpha }^{\left(0\right)}$ , and the softness potential $\nu_{Nu}^{s^{-}}\left(r_{p}\right)$ with its condensed form, $s_{Nu,\alpha }^{\left(-\right)}$ . These approximations, shown in eqs 13 and 15, generate the condensed Grand-Canonical General-Purpose Reactivity Indicator for each *α* nucleus in the nucleophile, $\Theta_{\Delta \mu \leq 0,\alpha }^{\kappa }$ ,

22 $$\Theta_{\Delta \mu \leq 0,\alpha }^{\kappa }=\left(\kappa +1\right)q_{Nu,\alpha }^{\left(0\right)}-\Delta \mu \left(\kappa -1\right)s_{Nu,\alpha }^{\left(-\right)}$$

Here, $s_{Nu,\alpha }^{\left(-\right)}$ is defined by using atomic charges as^43,69,92^

23 $$s_{Nu,\alpha }^{\left(-\right)}=Sf_{Nu,\alpha }^{\left(-\right)}=S\left(q_{Nu,\alpha }^{\left(-\right)}-q_{Nu,\alpha }^{\left(0\right)}\right)$$

In eq 23, $q_{Nu,\alpha }^{\left(0\right)}$ and $q_{Nu,\alpha }^{\left(-\right)}$ represent the atomic charges of each *α* atom in its ground state (typically a neutral species) and after the removal of one electron (typically a cation), respectively. The global softness (*S*) of a molecule was previously defined in eq 6 as $S={\left(\frac{\partial N}{\partial \mu }\right)}_{\nu \left(r\right)}$ ,^42,81^ which can be approximated using finite differences^32^ as the reciprocal of the energy gap between the first vertical ionization potential (*I*) and the first vertical electron affinity (*A*),

24 $$S=\frac{1}{I-A}$$

The values of *I* and *A* are computed using the ground state energy of the molecule, usually neutral, with *N* electrons (*E*(*N*)) and those of the species with one electron removed (*E*(*N* −1)) or added (*E*(*N* +1)), as follows,

25 I=E(N−1)−E(N)

26 A=E(N)−E(N+1)

Likewise, the GC-GPRI equation for electrophiles undergoing a nucleophilic attack arises directly from eq 12, Δ*U_int_*, employing the key parameter, ${\mathrm{\kappa ̃}}_{Nu}$ , to model the attacking nucleophile,

$${\mathrm{\kappa ̃}}_{Nu}\equiv -q_{Nu}^{\left(0\right)}-\Delta \mu s_{Nu}^{\left(-\right)}$$ 27

Thus, the resulting equation is the GC-GPRI for electrophiles undergoing a nucleophilic attack, $\Theta_{\Delta \mu \geq 0}^{\kappa }\left(r_{p}\right)$ ,

28 $$\Theta_{\Delta \mu \geq 0}^{\kappa }\left(r_{p}\right)=-\left(\kappa +1\right)\Phi_{Ele}\left(r_{p}\right)+\Delta \mu \left(\kappa -1\right)\nu_{Ele}^{s^{+}}\left(r_{p}\right)$$

As it is the case with eq 22, we can assign a GC-GPRI value for each nucleus *β* in the electrophile by replacing the electrostatic potential (Φ*_Ele_* (***r***_*p*_)) and the softness potential $\left(\nu_{Ele}^{s^{+}}\left(r_{p}\right)\right)$ by the atomic charges $\left(q_{Ele,\beta }^{\left(0\right)}\right)$ and the condensed local softness $\left(s_{Ele,\beta }^{\left(+\right)}\right)$ , respectively, to generate the condensed GC-GPRI for electrophiles, $\Theta_{\Delta \mu \geq 0,\beta }^{\kappa }$ ,

29 $$\Theta_{\Delta \mu \geq 0,\beta }^{\kappa }=-\left(\kappa +1\right)q_{Ele,\beta }^{\left(0\right)}+\Delta \mu \left(\kappa -1\right)s_{Ele,\beta }^{\left(+\right)}$$

In eq 29, $s_{Ele,\beta }^{\left(+\right)}$ represents the condensed local softness for each atom *β* in the electrophile,

30 $$s_{Ele,\beta }^{\left(+\right)}=Sf_{Ele,\beta }^{\left(+\right)}=S\left(q_{Ele,\beta }^{\left(0\right)}-q_{Ele,\beta }^{\left(+\right)}\right)$$

where $q_{Ele,\beta }^{\left(0\right)}$ and $q_{Ele,\beta }^{\left(+\right)}$ are the atomic charges of each *β* atom in the species with its neutral state and after accepting one electron, respectively.

## 3 Methodology

All calculations were performed using density-functional theory (DFT).^93–97^ Benchmark assessment the performance of the GC-GPRI for predicting molecular reactivity (as described in Section 4.1) is included in the Supporting Information. These benchmark calculations were carried out using the following functionals: (a) ωB97XD,^98^ (b) M062X,^99^ (c) PBE0,^100^ (d) PBE,^101^ (e) TPSS^102^ and (f) SVWN.^103^ Since all tested functionals yielded similar results in comparison to experimental reactivity trends (see Section 4 and Supporting Information), the B3LYP exchange-correlation energy functional^104–107^ combined with the 6–311++G** basis set^108–112^ was selected for all subsequent calculations. All computations were performed using the Gaussian 16 Rev. C software package.^113^ The neutral ground-state structures, *N* electron systems, were optimized, and single-point energy calculations were subsequently performed for the (*N*+1) and (*N*−1) electron systems at the optimized *N* electron geometry to compute energies and atomic charges. The Hirshfeld population scheme,^114^ recognized for its accuracy in computing condensed Fukui functions and GPRI values,^48,61^ was employed to ensure the reliable calculation of charge and the local softness, where global softness is generally insensitive to level of theory.^115–118^ In-house software was used to compute the values of the GC-GPRI as well as the Reactivity Transition Tables (RTTs) based on the original GPRI software.^60^

## 4 Results and Discussion

In this section, the outcomes of applying the condensed GC-GPRI to identify the reactive sites of nucleophiles and electrophiles undergoing electrophilic and nucleophilic attack, respectively, and to reproduce experimental nucleophilicity and electrophilicity scales are summarized.

### 4.1 Cases Studies: Elucidation of Reactive Sites in Molecules with Multiple Susceptible Atoms

Herein, both condensed GC-GPRI equations were employed to predict the reactivity of four molecules selected because of their complexity, as they contain multiple reactive sites. We computed the corresponding GC-GPRI equation for all atoms in each molecule. The results are presented in RTTs, which identify the atom in the molecule with the lowest GC-GPRI value for every combination of Δ*μ* and *κ*. The atom listed in each cell of the RTT is considered the most reactive site under the specific reaction conditions modeled by Δ*μ* and *κ*. These RTTs are similar to those used to showcase the predictions of the original GPRI, but with the parameter Δ*N* replaced by Δ*μ*, and Fukui functions by softness values.^40,54,90,119,120^ See Appendix A for the relationship between the GPRI and the GC-GPRI.

#### 4.1.1 Case Study 1 (CS1): Identifying Reactivity in Molecules with Multiple Nearly Identical Nucleophilic Sites

*N*,*N*,*N*′,*N″*,*N″-*Pentamethyldiethylenetriamine (PMDTA) is a flexible tridentate ligand that coordinates with metals through its three nitrogen atoms via lone pairs, see Scheme 1.^121–124^ All three nitrogen atoms are strong electron donors, but the central nitrogen (N2) is slightly more reactive than the terminal nitrogen atoms (both labeled as N1 due to their symmetry). Table 1 shows the results of the GC-GPRI for nucleophiles, which confirms that N2 is the most reactive site under most conditions while N1 is favored in pure electrostatic conditions, where (*κ* ≈ 1 and Δ*μ* ≈ 0). Table S1 shows that PBE, TPSS and SVWN functionals identified only N1 as the reactive site. Therefore, to ensure that GC-GPRI can effectively describe the reactivity of molecules with multiple reactive sites, we recommend using hybrid functionals with the GC-GPRI.

**Scheme 1:**
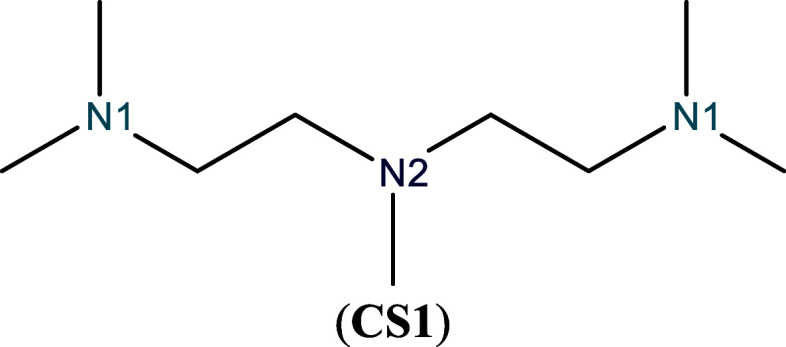
Molecular Representation of PMDTA

**Table 1:**
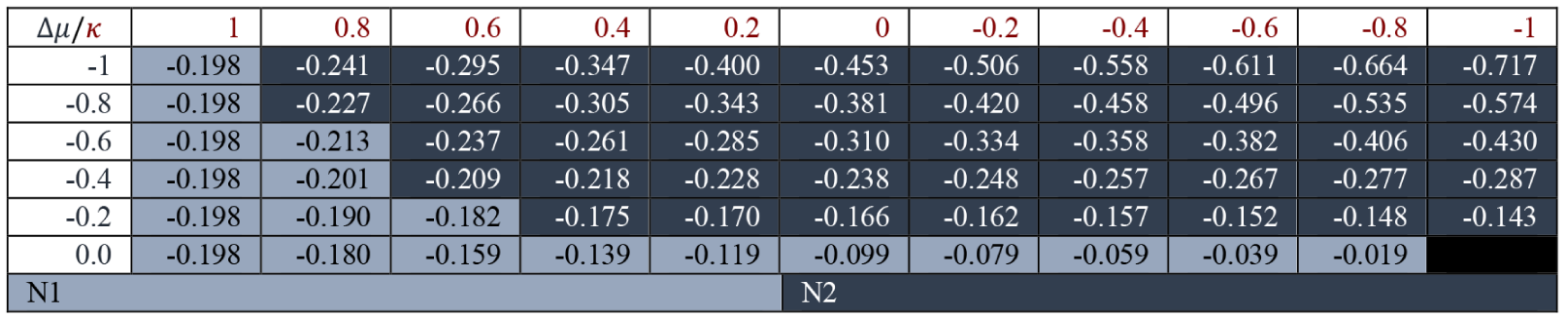
Reactivity transition table for the first choice of **CS1**, evaluated using the GC-GPRI for nucleophiles, (eq 22), $\Theta_{\Delta \mu \leq 0,\alpha }^{\kappa }=\left(\kappa +1\right)q_{Nu,\alpha }^{\left(0\right)}-\Delta \mu \left(\kappa -1\right)s_{Nu,\alpha }^{\left(-\right)}$ , and the Hirshfeld population scheme computed at the B3LYP/6–311++G** level of theory^a^

a The color of each cell indicates the most reactive atom under the reaction conditions given by Δ*μ* and *κ*. The values are reported in atomic units.

#### 4.1.2 Case Study 2 (CS2): Identifying the Most Reactive Atom in a Highly Delocalized System

Benzo­[*a*]­pyrene (**CS2**) is a highly delocalized polyaromatic hydrocarbon. However, despite this delocalization, experimental evidence clearly indicates that C6 is the most susceptible site for electrophilic attack.^125–127^ Electrochemical oxidation produced various byproducts, indicating the second most reactive site, with distributions at C1 (47%), C3 (33%) and C12 (20%),^125^ see Scheme 2. Due to its extensive Electron Delocalization (ED), **CS2** presents a challenging test case for the GC-GPRI model, as this approach does not explicitly account for ED effects. The RTTs for **CS2** shown in Table 2 correctly identify C6 (A) and C1 (B) as the most and second most reactive atoms, respectively, under both soft (electron-transfer) and hard (electrostatic) conditions. When the GC-GPRI is evaluated at purely electrostatic control conditions, *κ* = 1, C2 and C8 are predicted to be the first and second most reactive atoms, which deviate from experimental observations. Overall, the model correctly predicts the regioselectivity of the two major products in the experimental mixture.

**Scheme 2:**
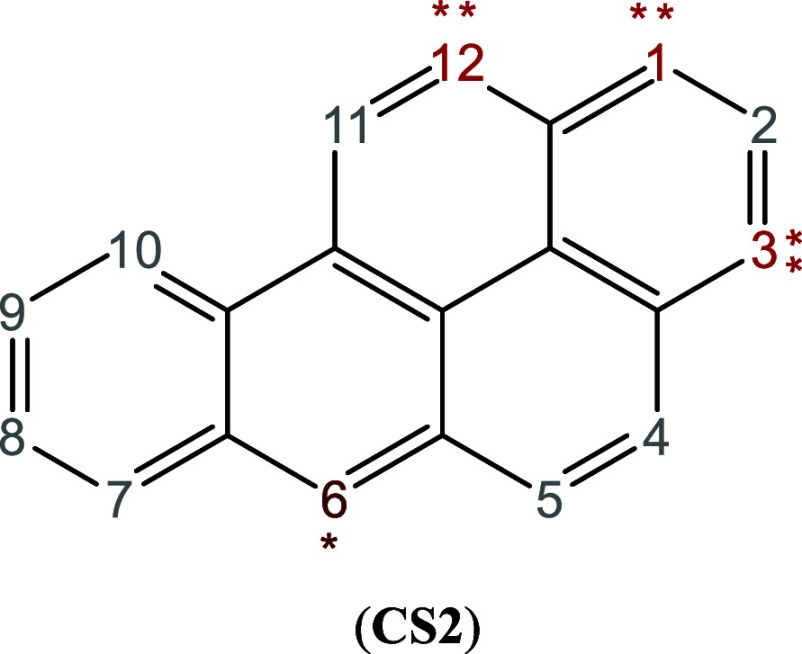
Molecular Representation of Benzene­[*a*]­pyrene[Fn sch2-fn1]

I The experimental most and second most reactive atoms are indicated with one and two asterisks, respectively

**Table 2:**
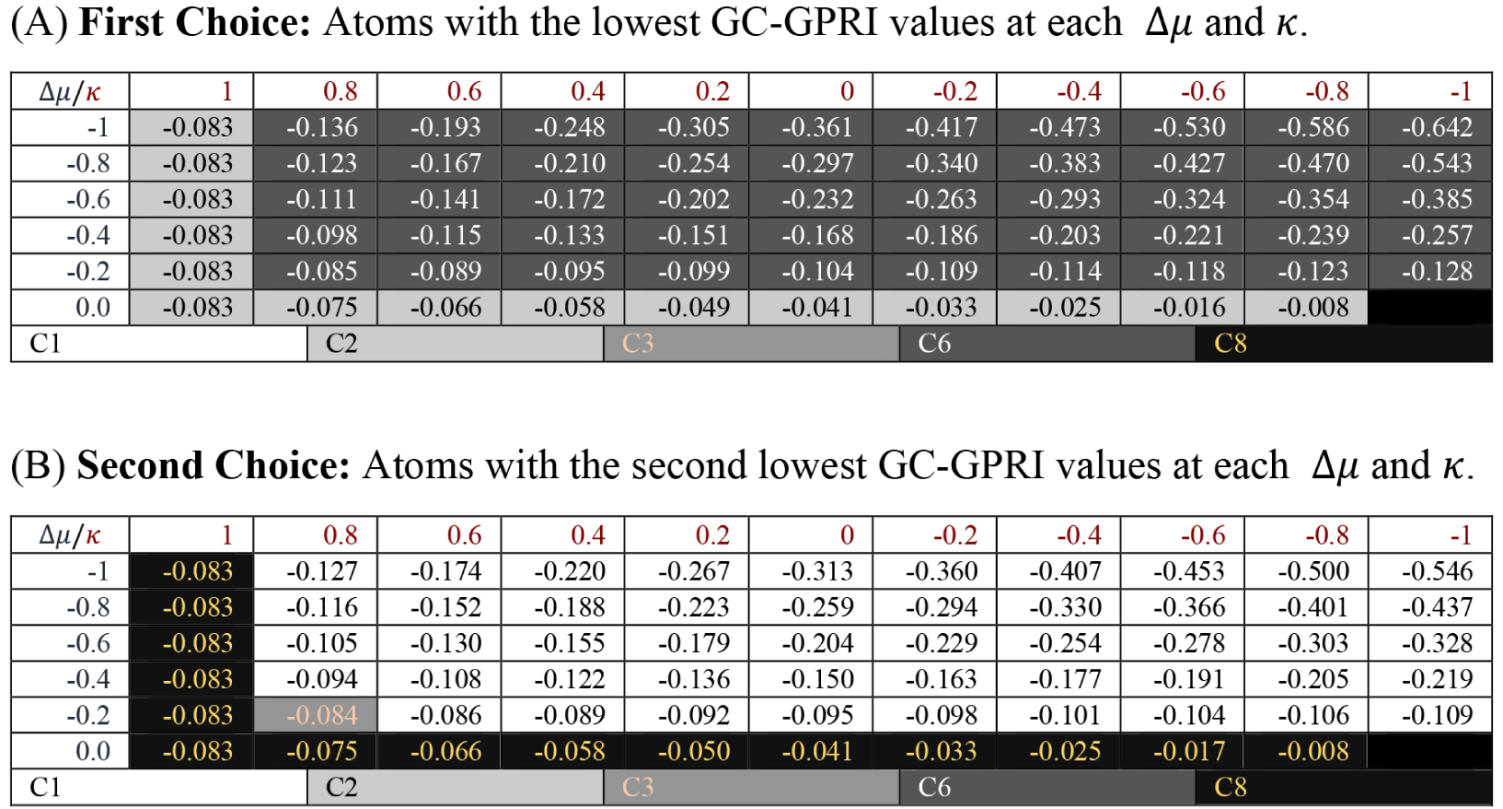
Reactivity transition table for **CS2**, using the GC-GPRI for nucleophiles, (eq 22), $\Theta_{\Delta \mu \leq 0,\alpha }^{\kappa }=\left(\kappa +1\right)q_{Nu,\alpha }^{\left(0\right)}-\Delta \mu \left(\kappa -1\right)s_{Nu,\alpha }^{\left(-\right)}$ , and the Hirshfeld population scheme computed at the B3LYP/6–311++G** level of theory^a^

a Sections (A) and (B) show the most and second most reactive atoms, respectively. The color of each cell indicates the most reactive atom under the reaction conditions given by Δ*μ* and *κ*. The values are reported in atomic units.

#### 4.1.3 Case Study 3 (CS3): Identifying Reactive Sites for Nucleophilic Addition in a Diiminium Adduct

The *N*-(tetramethylformamidinio)­pyridinium salt (**CS3**), Scheme 3, exhibits distinct experimental reactivities under different reaction conditions, with C4 and C2/C6 as the first two most reactive sites under soft conditions,^128,129^ while C1 reacts with hard fluoride anions.^128,129^ Table 3A reports the most reactive atom by the GC-GPRI analysis for **CS3**. Herein, the GC-GPRI highlighted C4 and C1 as the primary reactive sites, under soft and hard conditions, respectively. Table 3B accounts that the GC-GPRI predicts C2 as the second most reactive atom under soft conditions, in accordance with experimental observations.^66,130^

**Scheme 3:**
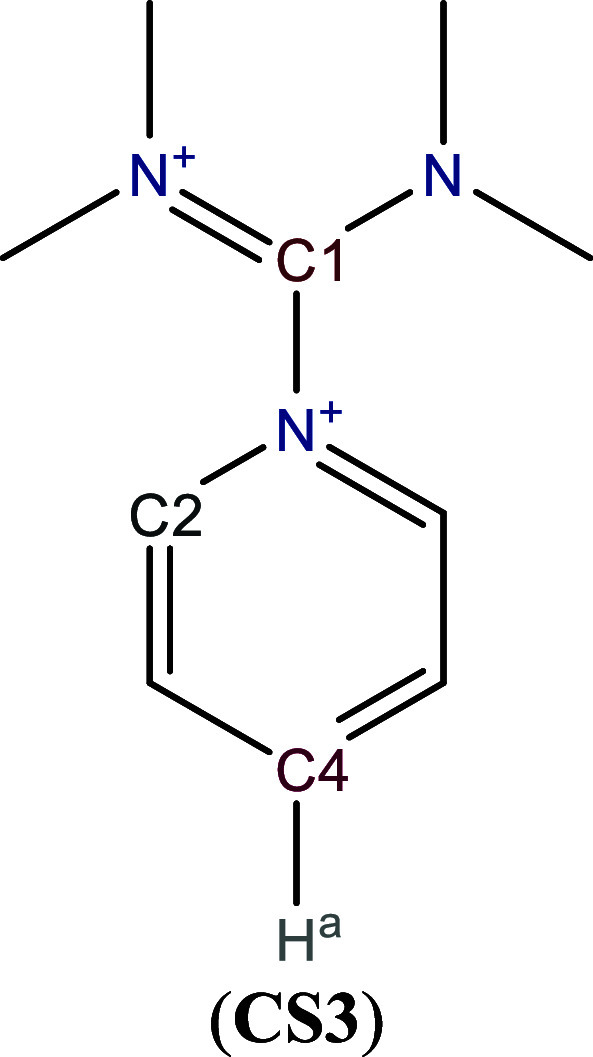
Molecular Representation of *N*-(tetramethylformamidinio)­pyridinium

**Table 3:**
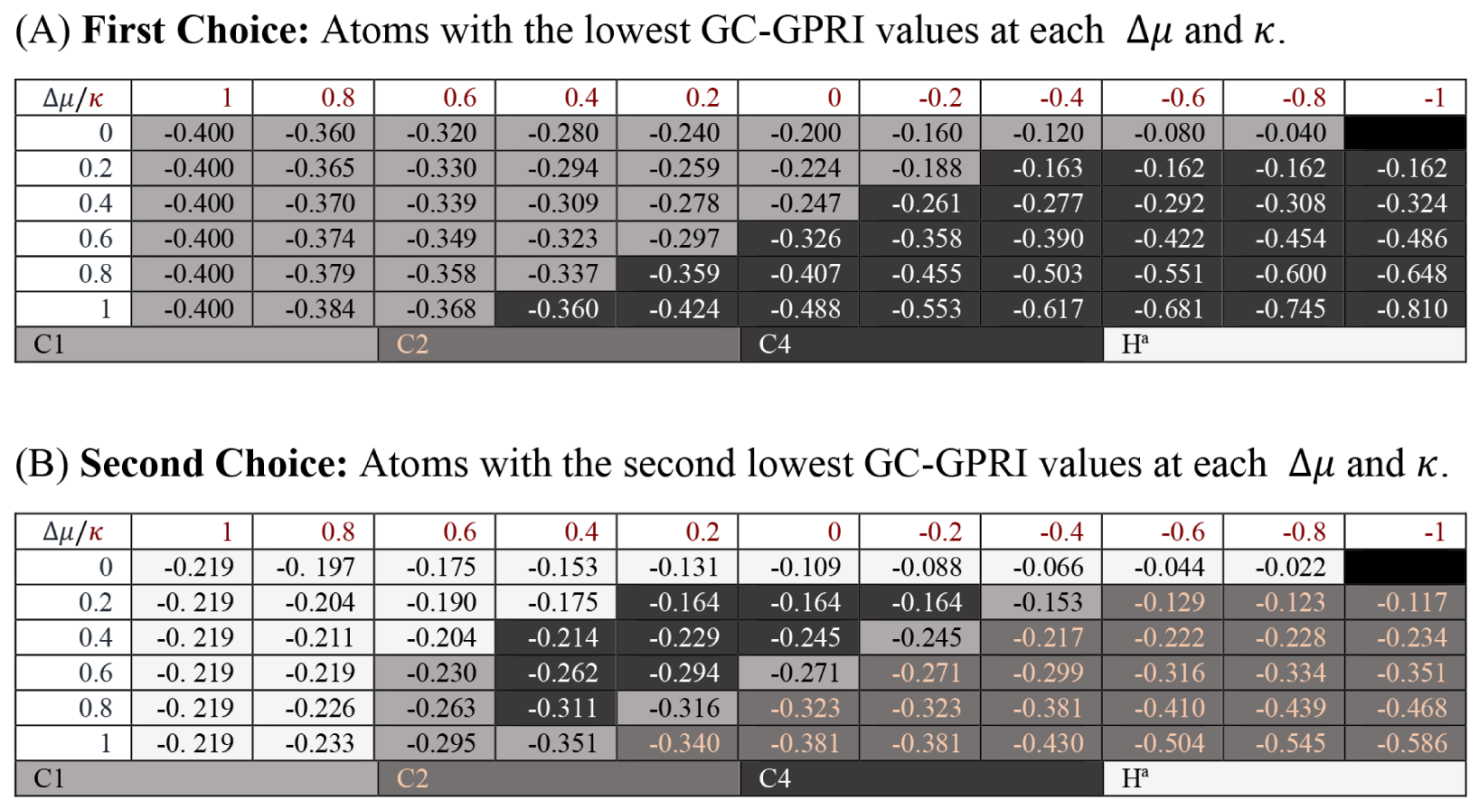
Reactivity transition table for **CS3**, using the GC-GPRI for electrophiles, (eq 29), $\Theta_{\Delta \mu \geq 0,\beta }^{\kappa }=-\left(\kappa +1\right)q_{Ele,\beta }^{\left(0\right)}+\Delta \mu \left(\kappa -1\right)s_{Ele,\beta }^{\left(+\right)}$ , and the Hirshfeld population scheme computed at the B3LYP/6–311++G** level of theory^a^

a Sections (A) and (B) show the most and second most reactive atoms, respectively. The color of each cell indicates the most reactive atom under the reaction conditions given by Δ*μ* and *κ*. The values are reported in atomic units.

#### 4.1.4 Case Study 4 (CS4): Nucleophilic Addition in Organic Synthesis

The 6-chloroimidazo­[1,2-*a*]­pyrazine (**CS4**), shown in Scheme 4, exhibits broad reactivity with different metal salts and reaction conditions.^131^ Knochel et al. identified C1 and C4 as the primary and secondary sites for nucleophilic attacks, respectively.^131^ The GC-GPRI results, Table 4A,B, confirm that C1 is the most reactive atom under most conditions (hard and soft). The attack on C1 is proposed to occur via a directed mechanism, initially forming an adduct at N8, which shares its nucleophilic double bond with C9. Under purely electrostatic control (Δ*μ* = 0 and *κ* = 1), C9 emerges as the most reactive site. Additionally, Table 4B depicts C4 as the second most reactive site under electron-transfer conditions, H^a^ and H^b^ for electrostatically controlled reactions. These results indicate that electrostatic interactions could play a crucial role in the initial approach of the attacking molecule. Particularly since H^a^, H^b^ and C9 are bonded to C1 and C4, the experimentally identified reactive sites.

**Scheme 4:**
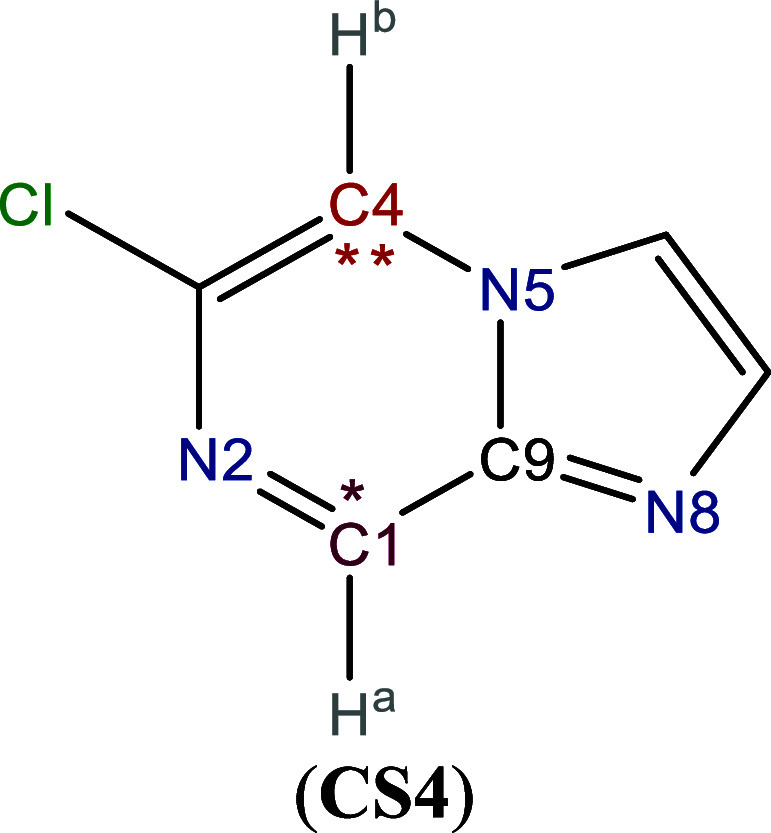
Molecular Representation of 6-chloroimidazo­[1,2-*a*]­pyrazine[Fn sch4-fn2]

i The most and second most reactive atoms for nucleophilic attack are marked with one and two asterisks, respectively

**Table 4:**
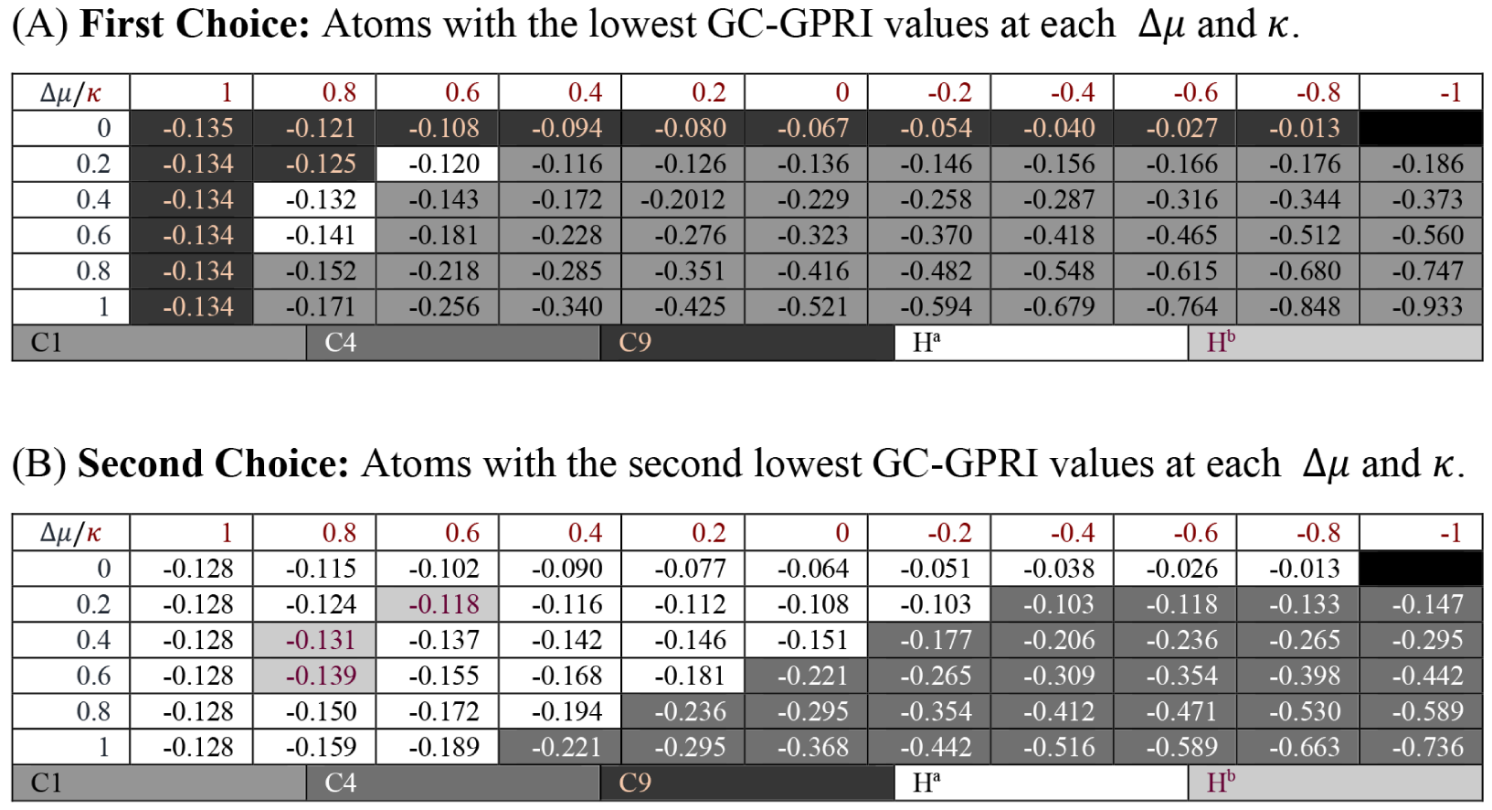
Reactivity transition table for **CS4**, using the GC-GPRI for electrophiles, eq 29, $\Theta_{\Delta \mu \geq 0,\beta }^{\kappa }=-\left(\kappa +1\right)q_{Ele,\beta }^{\left(0\right)}+\Delta \mu \left(\kappa -1\right)s_{Ele,\beta }^{\left(+\right)}$ , and the Hirshfeld population scheme computed at the B3LYP/6–311++G** level of theory^a^

a Sections (A) and (B) show the most and second most reactive atoms, respectively. The color of each cell indicates the most reactive atom under the reaction conditions given by Δ*μ* and *κ*. The values are reported in atomic units.

### 4.2 Performance of the Condensed GC-GPRI in the Reproduction of Experimental Nucleophilicity and Electrophilicity Scales

Now, we apply the GC-GPRI, evaluated at different reactivity conditions modeled by Δ*μ* and *κ* values, to reproduce the experimental nucleophilicity and electrophilicity scales of 41 molecules (20 nucleophiles and 21 electrophiles).

#### 4.2.1 Experimental Nucleophilicity Scale

Scheme 5 displays the molecular structure of the 20 nucleophiles of interest (**N1–N20**). The experimentally determined most reactive atom(s) in the corresponding molecule undergoing an electrophilic attack is (are) indicated with red stars.^47,48^

**Scheme 5:**
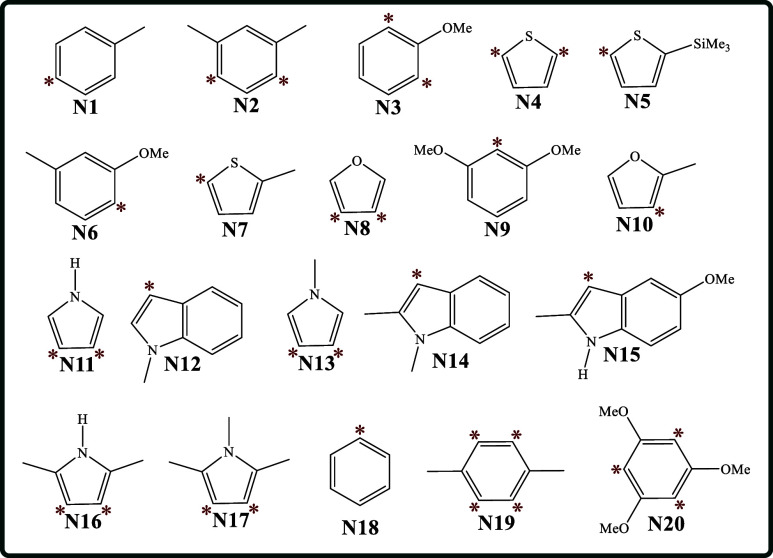
Molecular Structures of the 20 Nucleophiles (**N1–N20**) Examined to Reproduce the Experimental Nucleophilicity Scale[Fn sch5-fn3]

i The red stars indicate the experimentally most susceptible atoms to electrophilic attack.^2,48^

Figure 1 shows the correlations between experimental data and GC-GPRI values for nucleophiles, $\Theta_{\Delta \mu \leq 0,\alpha }^{\kappa }$ (eq 22), evaluated at the most reactive atoms (highlighted with red starts in Scheme 5) under five different reactivity scenarios (cases), labeled from (a) to (e). These conditions represent combinations of the parameters *κ* and Δ*μ*, capturing the transition from electrostatic to electron transfer controlled reactions, as explained below:

(a) Δ*μ* = 0 and *κ* = 0: pure electrostatic behavior, which reflects the exact atomic charge.
(b) Δ*μ* = −0.05 and *κ* = 0.8: strong electrostatic behavior with minor electron transfer.
(c) Δ*μ* = −0.5 and *κ* = 0: balanced electrostatic and electron transfer contributions.
(d) Δ*μ* = −0.95 and *κ* = 0.8: predominantly electron transfer with some electrostatic influence given by *κ* = 0.8.
(e) Δ*μ* = −0.95 and *κ* = −0.8: dominant electron transfer behavior, which corresponds to the softest condition evaluated.

**Figure 1:**
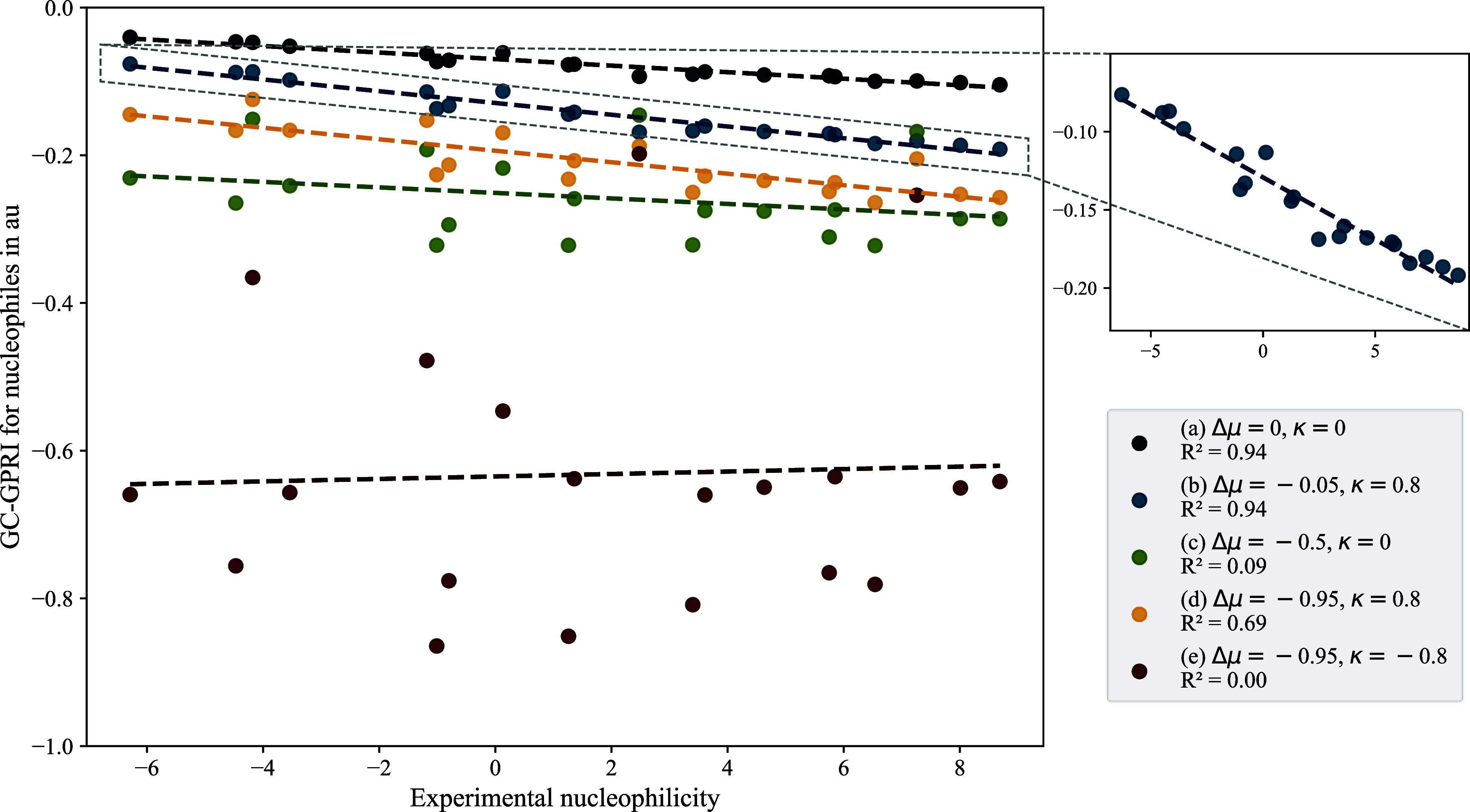
Correlations between experimental nucleophilicity (*N*) and the condensed GC-GPRI for the most reactive atoms of the nucleophiles shown in Scheme 5. The GC-GPRI values were computed using eq 22, $\Theta_{\Delta \mu \leq 0,\alpha }^{\kappa }=\left(\kappa +1\right)q_{Nu,\alpha }^{\left(0\right)}-\Delta \mu \left(\kappa -1\right)s_{Nu,\alpha }^{\left(-\right)}$ , and the Hirshfeld population scheme at the B3LYP/6–311++G** level of theory.

The *R* ^2^ values in Figure 1 show that GC-GPRI predictions are considerably sensitive to the chosen reactivity conditions. For example, a linear correlation between GC-GPRI and experimentally nucleophilicities is observed under electrostatically controlled conditions, cases (a) and (b). Figure 1 highlights the correlation found in case (b). A negative slope indicates an inverse relationship between experimental nucleophilicity and GC-GPRI values. Consequently, species with higher experimental nucleophilicity exhibit more negative $\Theta_{\Delta \mu \leq 0,\alpha }^{\kappa }$ values, which indicates a greater reactivity toward electrophilic attack. However, the lack of correlation observed under the extreme electron transfer condition used (Δ*μ* = −0.95 and *κ* = 0.8) reflects the fact that such regimes are not representative of typical nucleophilic reactivity, which is primarily governed by electrostatic interactions.^48,132^ When electron transfer is artificially increased in the GC-GPRI by adjusting Δ*μ*, its predictive power decreases, which leads to deviations from experimental nucleophilicity. This circumstance can be observed in options (c) and (e) of Figure 1, where the indicator does not fully capture the experimental trends.

The sensitivity of the calculated nucleophilicity using GC-GPRI to variations in reactivity conditions raises the question, “Within what reactivity range does the model best match experimental nucleophilicity scales?”. To address this inquiry, we varied Δ*μ* from 0 to −1 in six steps, (f) Δ*μ* = 0, (g) Δ*μ* = −0.2, (h) Δ*μ* = −0.4, (i) Δ*μ* = −0.6, (j) Δ*μ* = −0.8 and (*k*) Δ*μ* = −1, while keeping *κ* constant at 0.8. Figure 2 illustrates that as electron transfer increases, the *R* ^2^ values decrease, with the strongest linear relationship between computed and experimental data observed when Δ*μ* ranges from −0.4 to 0.

**Figure 2:**
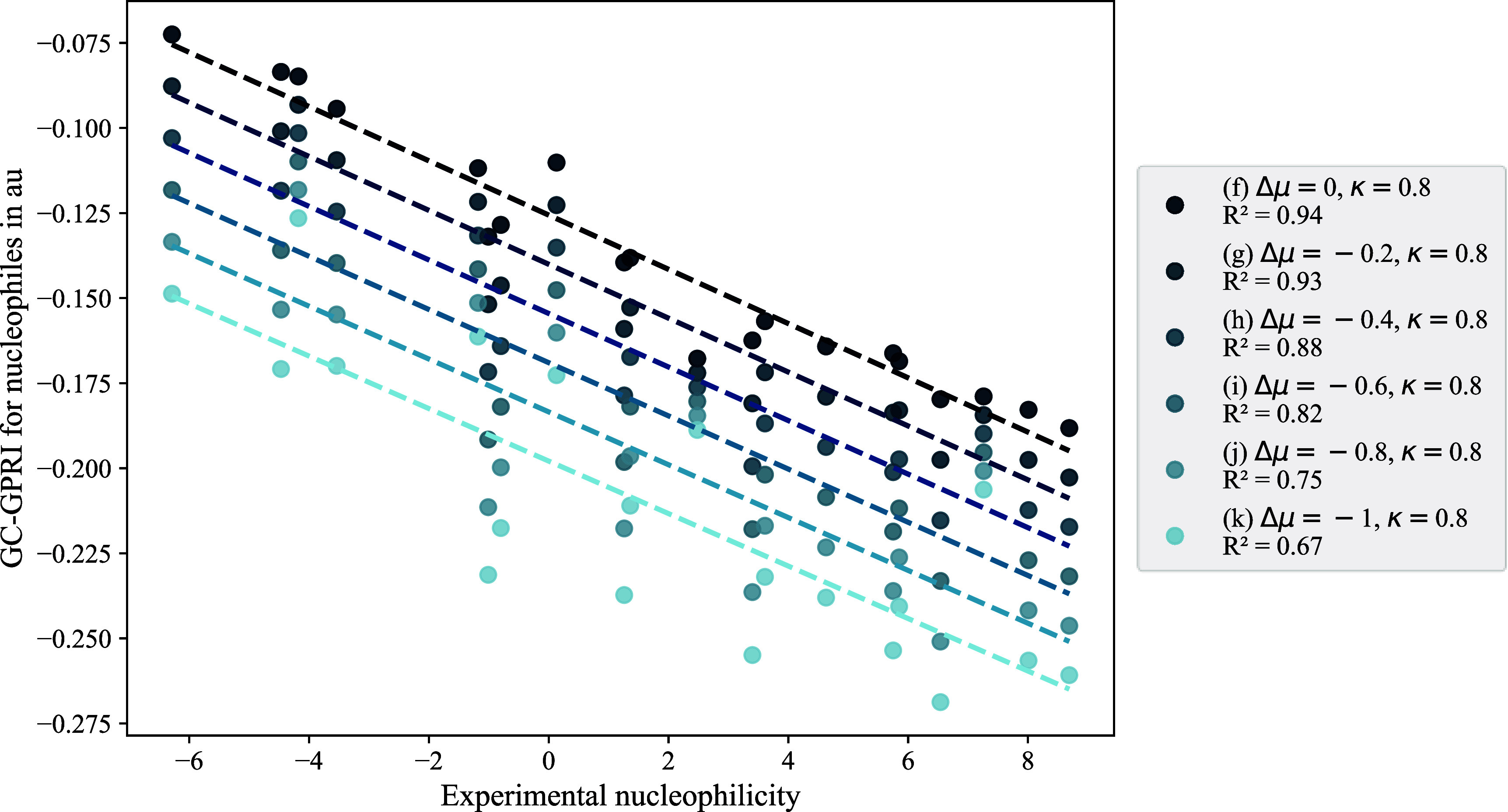
Correlations between experimental nucleophilicity (*N*) and the condensed GC-GPRI for the most reactive atoms of the nucleophiles shown in Scheme 5. The GC-GPRI values were computed using eq 22, $\Theta_{\Delta \mu \leq 0,\alpha }^{\kappa }=\left(\kappa +1\right)q_{Nu,\alpha }^{\left(0\right)}-\Delta \mu \left(\kappa -1\right)s_{Nu,\alpha }^{\left(-\right)}$ , and the Hirshfeld population scheme at the B3LYP/6–311++G** level of theory. The reactivity conditions considered Δ*μ* numbers from 0 to 1, keeping *κ* constant at 0.8.

#### 4.2.2 Experimental Electrophilicity Scale

Scheme 6 illustrates the molecular structure of the twenty-one electrophiles examined in this study (**E1–E21**). The red stars represent the most susceptible atom in the corresponding molecule undergoing a nucleophilic attack at different experimental reactivity conditions and electrophiles.^2,20,133,134^

**Scheme 6:**
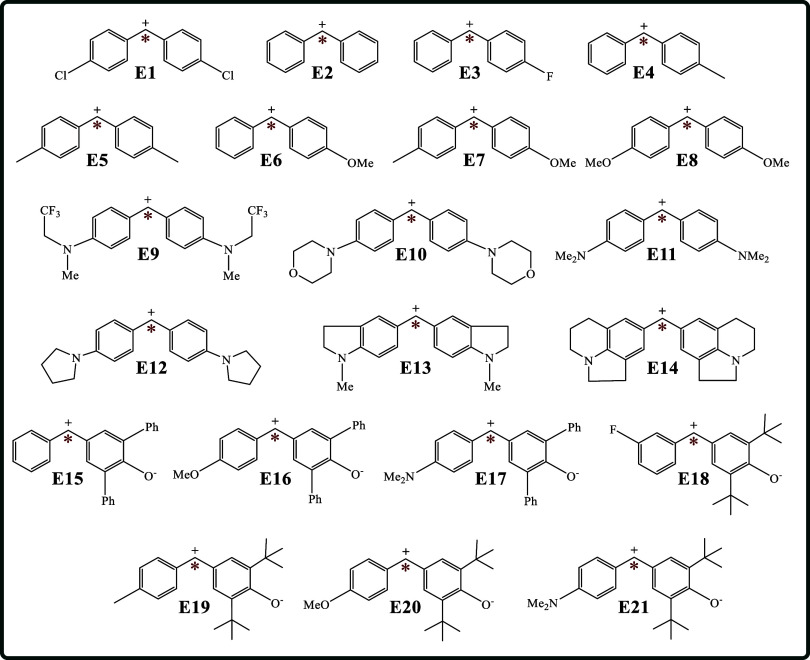
Molecular Structure of the Twenty-One Electrophiles Examined Herein to Reproduce an Electrophilicity Scale (**E1**-**E21**)­[Fn sch6-fn4]

i The red stars highlight the most susceptible atom to nucleophilic attack.^2,48^

Figure 3 shows the linear correlations between the GC-GPRI values and the experimental electrophilicity parameter (*E*).^2^ The modeled conditions mirror those used in the nucleophilic case described in Section 4, with the primary distinction being that in this case Δ*μ* is positive, which reflects the electron-accepting nature of electrophiles during nucleophilic attack. The *R* ^2^ values range from 0.82, observed under the strongest electron transfer control conditions, case (e), to 0.98 which corresponds to electrostatic control conditions, cases (a) and (b), and predominantly electron transfer with some electrostatic influence, case (d). These results underscore the capability of the GC-GPRI to accurately replicate experimental electrophilicity across a wide range of chemical environments.^135^ Figure 3 highlights case (b), which reveals an inverse correlation between GC-GPRI and experimental electrophilicity, indicating that the lower the GC-GPRI values correspond to lower interaction energy and stronger experimental electrophilicity behavior.

**Figure 3:**
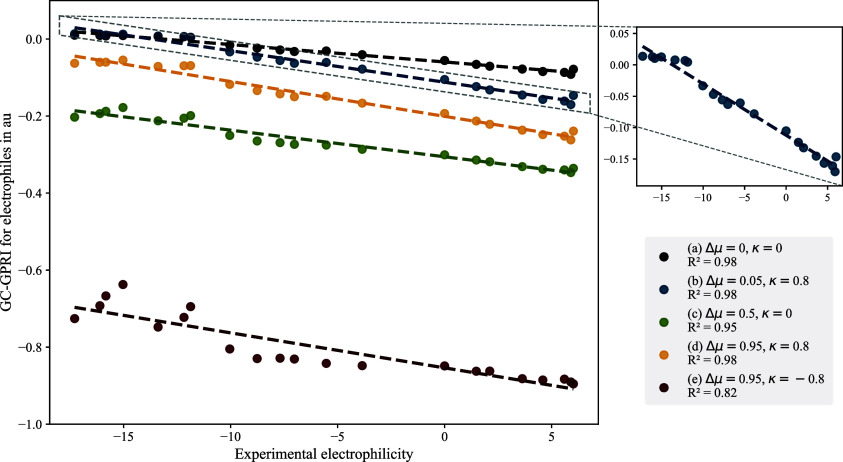
Correlations between experimental electrophilicity (*E*) and the condensed GC-GPRI for the most reactive atoms of the electrophiles shown in Scheme 6, using eq 29, $\Theta_{\Delta \mu \geq 0,\beta }^{\kappa }=-\left(\kappa +1\right)q_{Ele,\beta }^{\left(0\right)}+\Delta \mu \left(\kappa -1\right)s_{Ele,\beta }^{\left(+\right)}$ , and the Hirshfeld population scheme computed at the B3LYP/6–311++G** level of theory.

## 5 Conclusions

Herein, we developed and implemented the Grand Canonical General-Purpose Reactivity Indicator (GC-GPRI) which is a versatile model derived using a perturbative approach within the zero-temperature grand canonical ensemble of conceptual density-functional theory. It is designed to capture a wide range of reactivity conditions, which are modulated by the parameters Δ*μ* and *κ*. The condensed GC-GPRI enables the identification of the most reactive atoms in both nucleophiles and electrophiles including discerning, which atom is most likely to undergo hard or soft reactions. In all cases considered the GC-GPRI correctly discerned the first and second most reactive atoms within the hard and soft modes of reactivity. The proposed model successfully captures the reactivity of the studied set of molecules across various DFT functionals (Tables S1–S4 ). The only exception was the molecule in Case Study 1 (CS1), where the GC-GPRI model failed to resolve multiple reactive sites when using the PBE, TPSS and SVWN functionals. Therefore, we recommend using hybrid functionals, which provide the most reliable predictions. Our results are in line with the observation that the deviations in Hirshfeld charges and softness yield similar values over a variety of density functionals and basis sets.^115–118^ The GC-GPRI accurately reproduces experimental nucleophilicity and electrophilicity across diverse reactivity conditions. Moreover, under electrostatic control, the strong linear correlations depicted between computed GC-GPRI values and experimental electrophilicity and nucleophilicity yield *R* ^2^ values of 0.94 and 0.98. However, these high correlations are not consistent across all reactivity regimes, particularly those dominated by electron transfer, where the model exhibits reduced accuracy in reproducing experimental nucleophilicity and electrophilicity scales. Hence, the condensed GC-GPRI provides chemists with a robust reliable framework for predicting chemical reactivity and ranking nucleophiles and electrophiles.

## Supplementary Material



## Appendix A

### On the Δ*N* and Δ*μ* relationship between the GPRI and GC-GPRI

The condensed GPRI for nucleophiles and electrophiles is represented by eqs 31 and 32, respectively,^54^

31
$$\Xi_{\Delta N\leq 0,\alpha }^{\kappa }=\left(\kappa +1\right)q_{Nu,\alpha }^{\left(0\right)}-\Delta N\left(\kappa -1\right)f_{Nu,\alpha }^{\left(-\right)}$$

32
$$\Xi_{\Delta N\geq 0,\beta }^{\kappa }=-\left(\kappa +1\right)q_{Ele,\beta }^{\left(0\right)}+\Delta N\left(\kappa -1\right)f_{Ele,\beta }^{\left(+\right)}$$

The condensed GC-GPRI (eqs 22 and 29) can be rewritten in terms of the Fukui function and global softness (*S*) instead of the local softness for nucleophiles and electrophiles as shown in eqs 33 and 34,

33
$$\Theta_{\Delta \mu \leq 0,\alpha }^{\kappa }=\left(\kappa +1\right)q_{Nu,\alpha }^{\left(0\right)}-\Delta \mu \left(\kappa -1\right)S_{Nu}f_{Nu,\alpha }^{\left(-\right)}$$

34
$$\Theta_{\Delta \mu \geq 0,\beta }^{\kappa }=-\left(\kappa +1\right)q_{Ele,\beta }^{\left(0\right)}+\Delta \mu \left(\kappa -1\right)S_{Ele}f_{Ele,\beta }^{\left(+\right)}$$

There is an approximate relationship between the change in the number of electrons (labeled as Δ*N_GC_ _–GPRI_* in the GC-GPRI) and the change in chemical potential, mediated by global softness. This can be seen by comparing the second terms of the GPRI and GC-GPRI expression,^40^ similar to eq 21,

35
$$\Delta \mu \approx \Delta N_{GC-GPRI}\left(\frac{1}{S_{Nu}}+\frac{1}{S_{Ele}}\right)$$

The substitution of eq 35 into (eqs 33 and 34) yields,

36
$$\Theta_{\Delta \mu \leq 0,\alpha }^{\kappa }=\left(\kappa +1\right)q_{Nu,\alpha }^{\left(0\right)}-\Delta N_{GC-GPRI}\left(\frac{1}{S_{Nu}}+\frac{1}{S_{Ele}}\right)\left(\kappa -1\right)S_{Nu}f_{Nu,\alpha }^{\left(-\right)}$$

37
$$\Theta_{\Delta \mu \geq 0,\beta }^{\kappa }=-\left(\kappa +1\right)q_{Ele,\beta }^{\left(0\right)}+\Delta N_{GC-GPRI}\left(\frac{1}{S_{Nu}}+\frac{1}{S_{Ele}}\right)\left(\kappa -1\right)S_{Ele}f_{Ele,\beta }^{\left(+\right)}$$

These last two expressions can be reorganized as,

38
$$\Theta_{\Delta \mu \leq 0,\alpha }^{\kappa }=\left(\kappa +1\right)q_{Nu,\alpha }^{\left(0\right)}-\Delta N_{GC-GPRI}\left(1+\frac{S_{Nu}}{S_{Ele}}\right)\left(\kappa -1\right)f_{Nu,\alpha }^{\left(-\right)}$$

39
$$\Theta_{\Delta \mu \geq 0,\beta }^{\kappa }=-\left(\kappa +1\right)q_{Ele,\beta }^{\left(0\right)}+\Delta N_{GC-GPRI}\left(1+\frac{S_{Ele}}{S_{Nu}}\right)\left(\kappa -1\right)f_{Ele,\beta }^{\left(+\right)}$$

To compare how electron number changes are treated in GPRI and GC-GPRI, we examine eqs 31 and 38 for nucleophiles, and eqs 32 and 39 for electrophiles. The following expressions highlight their differences

40
$$\Delta N=\Delta N_{GC-GPRI}\left(1+\frac{S_{Nu}}{S_{Ele}}\right)$$

41
$$\Delta N=\Delta N_{GC-GPRI}\left(1+\frac{S_{Ele}}{S_{Nu}}\right)$$

for nucleophiles and electrophiles, respectively. Note that the Δ*N* from eq 21 is often considered small, having absolute values, |Δ*N*| < 0.4.^40,91,136–138^ Since softness and hardness are positive, then the value of Δ*N_GC_ _–GPRI_* for nucleophiles in eq 40 is enhanced by the factor $\left(1+\frac{S_{Nu}}{S_{Ele}}\right)$ and likewise Δ*N_GC_ _–GPRI_* for electrophiles in eq 41 is enhanced by the factor $\left(1+\frac{S_{Ele}}{S_{Nu}}\right)$ .

## Notes

The condensed GC-GPRI Reactivity Transition Tables (RTTs) containing predictions of the most reactive atoms in the molecules in Section 4 computed using the basis set 6–311++G**, the Hirshfeld population scheme, and the DFT methods: (a) ωB97XD, (b) M062X, (c) PBE0, (d) PBE, (e) TPSS, and (f) SVWN (PDF)

## References

[1] Ingold C. K. (1934). Principles
of an Electronic Theory of Organic Reactions. Chem. Rev..

[2] Mayr H., Patz M. (1994). Scales of Nucleophilicity and Electrophilicity: A System for Ordering
Polar Organic and Organometallic Reactions. Angew. Chem., Int. Ed..

[3] Allgäuer D. S., Jangra H., Asahara H., Li Z., Chen Q., Zipse H., Ofial A. R., Mayr H. (2017). Quantification and
Theoretical Analysis of the Electrophilicities of Michael Acceptors. J. Am. Chem. Soc..

[4] Lemek T., Mayr H. (2003). Electrophilicity Parameters
for Benzylidenemalononitriles. J. Org. Chem..

[5] Contreras R.
R., Fuentealba P., Galván M., Pérez P. (1999). A Direct Evaluation
of Regional Fukui Functions in Molecules. Chem.
Phys. Lett..

[6] Geerlings P., Chamorro E., Chattaraj P. K., De Proft F., Gázquez J. L., Liu S., Morell C., Toro-Labbe A., Vela A., Ayers P. (2020). Conceptual
Density Functional Theory: Status, Prospects, Issues. Theor. Chem. Acc..

[7] Gázquez J. L., Cedillo A., Vela A. (2007). Electrodonating
and Electroaccepting
Powers. J. Phys. Chem. A.

[8] Morell C., Gázquez J. L., Vela A., Guégan F., Chermette H. (2014). Revisiting
Electroaccepting and Electrodonating Powers:
Proposals for Local Electrophilicity and Local Nucleophilicity Descriptors. Phys. Chem. Chem. Phys..

[9] Pal R., Chattaraj P. K. (2023). Electrophilicity
Index Revisited. J. Comput. Chem..

[10] De
Vleeschouwer F., Van Speybroeck V., Waroquier M., Geerlings P., De Proft F. (2007). Electrophilicity and Nucleophilicity
Index for Radicals. Org. Lett..

[11] Jaramillo P., Pérez P., Contreras R., Tiznado W., Fuentealba P. (2006). Definition
of a Nucleophilicity Scale. J. Phys. Chem. A.

[12] O’Hara F., Blackmond D. G., Baran P. S. (2013). Radical-Based Regioselective C–H
Functionalization of Electron-Deficient Heteroarenes: Scope, Tunability,
and Predictability. J. Am. Chem. Soc..

[13] O’Brien A. G., Maruyama A., Inokuma Y., Fujita M., Baran P. S., Blackmond D. G. (2014). Radical
C-H Functionalization of Heteroarenes under
Electrochemical Control. Angew. Chem., Int.
Ed..

[14] Erdmann P., Greb L. (2022). What Distinguishes the Strength and the Effect of a Lewis Acid: Analysis
of the Gutmann–Beckett Method. Angew.
Chem., Int. Ed..

[15] Swain C. G., Scott C. B. (1953). Quantitative Correlation of Relative Rates. Comparison
of Hydroxide Ion with Other Nucleophilic Reagents toward Alkyl Halides,
Esters, Epoxides and Acyl Halides. J. Am. Chem.
Soc..

[16] Ritchie C. D. (1972). Nucleophilic
Reactivities toward Cations. Acc. Chem. Res..

[17] Ritchie C. D., Sawada M. (1977). Cation-Anion Combination Reactions. 15. Rates of Nucleophilic
Aromatic Substitution Reactions in Water and Methanol Solutions. J. Am. Chem. Soc..

[18] Alavosus T. J., Sweigart D. A. (1985). Comparative Study
of Nucleophilic Addition to Free
and Metal-Coordinated Carbocations. J. Am. Chem.
Soc..

[19] Burfeindt J., Patz M., Müller M., Mayr H. (1998). Determination of the
Nucleophilicities of Silyl and Alkyl Enol Ethers. J. Am. Chem. Soc..

[20] Phan T. B., Breugst M., Mayr H. (2006). Towards a
General Scale of Nucleophilicity?. Angew. Chem.,
Int. Ed..

[21] Brotzel F., Chu Y. C., Mayr H. (2007). Nucleophilicities of Primary and
Secondary Amines in Water. J. Org. Chem..

[22] Hagen G., Mayr H. (1991). Kinetics of the Reactions
of Allylsilanes, Allylgermanes, and Allylstannanes
with Carbenium Ions. J. Am. Chem. Soc..

[23] Brotzel F., Mayr H. (2007). Nucleophilicities of Amino Acids and Peptides. Org. Biomol. Chem..

[24] Appel R., Mayr H. (2011). Quantification of the
Electrophilic Reactivities of Aldehydes, Imines,
and Enones. J. Am. Chem. Soc..

[25] Mayr H., Kempf B., Ofial A. R. (2003). π-Nucleophilicity
in Carbon–carbon
Bond-Forming Reactions. Acc. Chem. Res..

[26] Ree N., Wollschläger J. M., Göller A. H., Jensen J. H. (2025). Atom-Based Machine Learning for Estimating
Nucleophilicity
and Electrophilicity with Applications to Retrosynthesis and Chemical
Stability. Chem. Sci..

[27] Liu Y., Yang Q., Cheng J., Zhang L., Luo S., Cheng J.-P. (2023). Prediction of Nucleophilicity
and Electrophilicity
Based on a Machine-Learning Approach. ChemPhyschem.

[28] Hoffmann G., Balcilar M., Tognetti V., Héroux P., Gaüzère B., Adam S., Joubert L. (2020). Predicting
Experimental Electrophilicities from Quantum and Topological Descriptors:
A Machine Learning Approach. J. Comput. Chem..

[29] Pereira F., Latino D. A. R. S., Aires-de-Sousa J. (2011). Estimation of Mayr Electrophilicity
with a Quantitative Structure–Property Relationship Approach
Using Empirical and DFT Descriptors. J. Org.
Chem..

[30] Cuesta S. A., Moreno M., López R. A., Mora J. R., Paz J. L., Márquez E. A. (2023). ElectroPredictor: An Application to Predict Mayr’s
Electrophilicity E through Implementation of an Ensemble Model Based
on Machine Learning Algorithms. J. Chem. Inf.
Model..

[31] Geerlings P., De Proft F., Langenaeker W. (2003). Conceptual
Density Functional Theory. Chem. Rev..

[32] Chermette H. (1999). Chemical Reactivity
Indexes in Density Functional Theory. J. Comput.
Chem..

[33] Geerlings P., De Proft F. (2008). Conceptual DFT: The
Chemical Relevance of Higher Response
Functions. Phys. Chem. Chem. Phys..

[34] Proft, F. D. ; Ayers, P. W. ; Geerlings, P. The Conceptual Density Functional Theory Perspective of Bonding. The Chemical Bond: Fundamental Aspects of Chemical Bonding; Wiley Online Library, 2014, 233–270. 10.1002/9783527664696.ch7.

[35] Parr R. G., Levy M., Donnelly R. A., Palke W. E. (1978). Electronegativity:
The Density Functional Viewpoint. J. Chem. Phys..

[36] Mortier W.
J., Ghosh S. K., Shankar S. (1986). Electronegativity-Equalization Method
for the Calculation of Atomic Charges in Molecules. J. Am. Chem. Soc..

[37] Cardenas-Jiron G. I., Gutierrez-Oliva S., Melin J., Toro-Labbe A. (1997). Relations
between Potential Energy, Electronic Chemical Potential, and Hardness
Profiles. J. Phys. Chem. A.

[38] Chattaraj P. K. (1996). The Maximum
Hardness Principle: An Overview. Proc. Indian
Natl. Sci. Acad., Part A.

[39] Chattaraj P. K., Liu G. H. (1995). The Maximum Hardness Principle in the Gyftopoulos-Hatsopoulos
Three-Level Model for an Atomic or Molecular Species and Its Positive
and Negative Ions. Chem. Phys. Lett..

[40] Pearson R. G. (1988). Absolute
Electronegativity and Hardness: Application to Inorganic Chemistry. Inorg. Chem..

[41] Pearson R. G. (1999). Maximum
Chemical and Physical Hardness. J. Chem. Edu..

[42] De
Proft F., Liu S., Parr R. G. (1997). Chemical Potential,
Hardness, Hardness and Softness Kernel and Local Hardness in the Isomorphic
Ensemble of Density Functional Theory. J. Chem.
Phys..

[43] Berkowitz M., Parr R. G. (1988). Molecular Hardness and Softness, Local Hardness and
Softness, Hardness and Softness Kernels, and Relations among These
Quantities. J. Chem. Phys..

[44] Nalewajski R. F., Korchowiec J., Zhou Z. (1988). Molecular Hardness and Softness Parameters
and Their Use in Chemistry. Int. J. Quantum
Chem..

[45] York D. M., Yang W. (1996). A Chemical Potential Equalization Method for Molecular Simulations. J. Chem. Phys..

[46] Ayers P. W., Anderson J. S. M., Bartolotti L. J. (2005). Perturbative
Perspectives on the
Chemical Reaction Prediction Problem. Int. J.
Quantum Chem..

[47] Liu S., Rong C., Lu T. (2014). Information Conservation Principle
Determines Electrophilicity, Nucleophilicity, and Regioselectivity. J. Phys. Chem. A.

[48] Wang B., Rong C., Chattaraj P. K., Liu S. (2019). A Comparative Study
to Predict Regioselectivity, Electrophilicity and Nucleophilicity
with Fukui Function and Hirshfeld Charge. Theor.
Chem. Acc..

[49] Parr R. G., Yang W. (1984). Density Functional
Approach to the Frontier-Electron Theory of Chemical
Reactivity. J. Am. Chem. Soc..

[50] Murray J. S., Politzer P. (2011). The Electrostatic Potential: An Overview. Wiley Interdiscip. Rev.: Comput. Mol. Sci..

[51] Cedillo A., Contreras R., Galvan M., Aizman A., Andrés J., Safont V. S. (2007). Nucleophilicity Index from Perturbed Electrostatic
Potentials. J. Phys. Chem. A.

[52] Fukui K. (1982). Role of Frontier
Orbitals in Chemical Reactions. Science.

[53] Yu J., Su N. Q., Yang W. (2022). Describing
Chemical Reactivity with
Frontier Molecular Orbitalets. JACS Au..

[54] Anderson J. S. M., Ayers P. W., Melin J. (2007). Conceptual Density-Functional Theory
for General Chemical Reactions, Including Those That Are Neither Charge-nor
Frontier-Orbital-Controlled. 1. Theory and Derivation of a General-Purpose
Reactivity Indicator. J. Chem. Theory Comput..

[55] Anderson J.
S. M., Ayers P. W., Melin J. (2007). Conceptual Density-Functional Theory
for General Chemical Reactions, Including Those That Are Neither Charge-nor
Frontier-Orbital-Controlled. 2. Application to Molecules Where Frontier
Molecular Orbital Theory Fails. J. Chem. Theory
Comput..

[56] Anderson J. S. M., Ayers P. W. (2007). Predicting the Reactivity of Ambidentate Nucleophiles
and Electrophiles Using a Single, General-Purpose, Reactivity Indicator. Phys. Chem. Chem. Phy.s.

[57] Anderson J. S. M., Melin J., Ayers P. W. (2016). Using the
General-Purpose Reactivity
Indicator: Challenging Examples. J. Mol. Model..

[58] Anderson J. S. M., Liu Y., Thomson J. W., Ayers P. W. (2010). Predicting the Quality
of Leaving Groups in Organic Chemistry: Tests against Experimental
Data. J. Mol. Struct.: THEOCHEM..

[59] Barrera, Y. ; Anderson, J. S. M. Predicting Reactivity with a General-Purpose Reactivity Indicator. Chemical Reactivity: vol. 2: Approaches and Applications; Kaya, S. ; VonSzentpaly, L. ; Serdaroglu, G. ; Guo, L. , Eds.; Elsevier, 2023, pp. 159–180.

[60] Anderson J. S. M., Ayers P. W. (2014). Resolving the Nature
of the Reactive Sites of Phenylsulfinate
(PhSO_2_
^–^) with a Single General-Purpose
Reactivity Indicator. Comput. Theor. Chem..

[61] Barrera Y., Anderson J. S. M. (2024). Does the Radical
GPRI Strongly Depend on the Population
Scheme? A Comparative Study to Predict Radical Attack on Unsaturated
Molecules with the Radical General-purpose Reactivity Indicator. J. Comput. Chem..

[62] Contreras-Torres F. F., Basiuk V. A., Basiuk E. V. (2008). Regioselectivity
in Azahydro [60]
Fullerene Derivatives: Application of General-Purpose Reactivity Indicators. J. Phys. Chem. A.

[63] Wang H., Dong W., Shi J. (2023). Theoretical
Speculation on the Chemical
Reaction Activity Site and Degradation Mechanism of Chloramphenicol. Chem. Phys. Lett..

[64] Morell C., Toro-Labbe A. (2005). New Dual Descriptor for Chemical Reactivity. J. Phys. Chem. A.

[65] Cárdenas C., Rabi N., Ayers P. W., Morell C., Jaramillo P., Fuentealba P. (2009). Chemical Reactivity Descriptors for Ambiphilic Reagents:
Dual Descriptor, Local Hypersoftness, and Electrostatic Potential. J. Phys. Chem. A.

[66] Mulks F. F. (2024). Hard and
Soft Electrons and Holes. Chem.

[67] Stuyver T., Shaik S. (2020). Unifying Conceptual
Density Functional and Valence Bond Theory: The
Hardness–Softness Conundrum Associated with Protonation Reactions
and Uncovering Complementary Reactivity Modes. J. Am. Chem. Soc..

[68] Baekelandt B. G., Cedillo A., Parr R. G. (1995). Reactivity
Indices and Fluctuation
Formulas in Density Functional Theory: Isomorphic Ensembles and a
New Measure of Local Hardness. J. Chem. Phys..

[69] Roy R. K., Krishnamurti S., Geerlings P., Pal S. (1998). Local Softness and
Hardness Based Reactivity Descriptors for Predicting Intra-and Intermolecular
Reactivity Sequences: Carbonyl Compounds. J.
Phys. Chem. A.

[70] Cárdenas C., Ayers P. W., Cedillo A. (2011). Reactivity
Indicators for Degenerate
States in the Density-Functional Theoretic Chemical Reactivity Theory. J. Chem. Phys..

[71] Von
Lilienfeld O. A., Tuckerman M. E. (2006). Molecular Grand-Canonical Ensemble
Density Functional Theory and Exploration of Chemical Space. J. Chem. Phys..

[72] Gázquez, J. L. ; Franco-Pérez, M. ; Ayers, P. W. ; Vela, A. Conceptual Density Functional Theory in the Grand Canonical Ensemble Chemical Reactivity In Confined Systems: Theory, Modelling And Applications Wiley Online Library 2021 191–211 10.1002/9781119683353.ch11

[73] Parr R. G., Gazquez J. L. (1993). Hardness Functional. J. Phys.
Chem..

[74] Woolley R. G., Sutcliffe B. T. (1977). Molecular Structure and the Born-Oppenheimer Approximation. Chem. Phys. Lett..

[75] Liu S., Parr R. G. (1997). Second-Order Density-Functional
Description of Molecules
and Chemical Changes. J. Chem. Phys..

[76] Nalewajski R. F., Parr R. G. (1982). Legendre
Transforms and Maxwell Relations in Density
Functional Theory. J. Chem. Phys..

[77] Wang B., Geerlings P., Heidar-Zadeh F., Ayers P. W., De Proft F. (2025). Exploring
Intrinsic Bond Properties with the Fukui Matrix from Conceptual Density
Matrix Functional Theory. J. Chem. Theory Comput..

[78] Johnson, P. A. ; Bartolotti, L. J. ; Ayers, P. W. ; Fievez, T. ; Geerlings, P. Charge Density and Chemical Reactions: A Unified View from Conceptual DFT. In Modern charge-density analysis, C, G. ; P, M. , Ed.; Springer: Dordrecht, 2011, pp. 715–764.

[79] Ayers P. W., Parr R. G. (2000). Variational Principles for Describing Chemical Reactions:
The Fukui Function and Chemical Hardness Revisited. J. Am. Chem. Soc..

[80] Ayers, P. W. ; Yang, W. ; Bartolotti, L. J. Chattaraj, P. K. Fukui Function. In Chemical reactivity theory: A density functional view; CRC Press Taylor & Francis Group: Boca Raton, 2009; pp. 255–267

[81] Yang W., Parr R. G. H. (1985). Softness, and
the Fukui Function in the Electronic
Theory of Metals and Catalysis. Proc. Natl.
Acad. Sci. U. S. A..

[82] Ayers P. W., Parr R. G. (2001). Variational Principles
for Describing Chemical Reactions.
Reactivity Indices Based on the External Potential. J. Am. Chem. Soc..

[83] Sanderson R. T. (1951). An Interpretation
of Bond Lengths and a Classification of Bonds. Science.

[84] Berkowitz M. (1987). Density Functional
Approach to Frontier Controlled Reactions. J.
Am. Chem. Soc..

[85] Hammond G. S. (1955). A Correlation
of Reaction Rates. J. Am. Chem. Soc..

[86] Farcasiu D. (1975). The Use and
Misuse of the Hammond Postulate. J. Chem. Edu..

[87] Ayers P. W., Liu S., Li T. (2011). Stability
Conditions for Density Functional Reactivity
Theory: An Interpretation of the Total Local Hardness. Phys. Chem. Chem. Phys..

[88] Pearson R. G. (2005). Chemical
Hardness and Density Functional Theory. J. Chem.
Sci..

[89] Furtado J., De Proft F., Geerlings P. (2015). The Noble
Gases: How Their Electronegativity
and Hardness Determines Their Chemistry. J.
Phys. Chem. A.

[90] Parr R. G., Pearson R. G. (1983). Absolute Hardness:
Companion Parameter to Absolute
Electronegativity. J. Am. Chem. Soc..

[91] Pearson R. G. (1989). Absolute
Electronegativity and Hardness: Applications to Organic Chemistry. J. Org. Chem..

[92] Chandra A. K., Nguyen M. T. (2002). Use of Local Softness
for the Interpretation of Reaction
Mechanisms. Int. J. Mol. Sci..

[93] Hohenberg P., Kohn W. (1964). Inhomogeneous Electron
Gas. Phys. Rev..

[94] Levy M. (2010). On the Simple
Constrained-search Reformulation of the Hohenberg–Kohn Theorem
to Include Degeneracies and More (1964–1979). Int. J. Quantum Chem..

[95] Kohn W., Sham L. J. (1965). Self-Consistent
Equations Including Exchange and Correlation
Effects. Phys. Rev..

[96] Levy M. (1979). Universal
Variational Functionals of Electron Densities, First-Order Density
Matrices, and Natural Spin-Orbitals and Solution of the v-Representability
Problem. Proc. Natl. Acad. Sci. U. S. A..

[97] Lieb E. H. (1983). Density
Functionals for Coulomb Systems. Int. J. Quantum
Chem..

[98] Chai J.-D., Head-Gordon M. (2008). Long-Range Corrected Hybrid Density Functionals with
Damped Atom–Atom Dispersion Corrections. Phys. Chem. Chem. Phys..

[99] Zhao Y., Truhlar D. G. (2008). The M06 Suite of Density Functionals for Main Group
Thermochemistry, Thermochemical Kinetics, Noncovalent Interactions,
Excited States, and Transition Elements: Two New Functionals and Systematic
Testing of Four M06-Class Functionals and 12 Other Function. Theor. Chem. Acc..

[100] Adamo C., Barone V. (1999). Toward Reliable Density
Functional
Methods without Adjustable Parameters: The PBE0Model. J. Chem. Phys..

[101] Perdew J. P., Burke K., Ernzerhof M. (1996). Generalized
Gradient Approximation Made Simple. Phys. Rev.
Lett..

[102] Tao J., Perdew J. P., Staroverov V. N., Scuseria G. E. (2003). Climbing the Density
Functional Ladder: Nonempirical Meta–Generalized Gradient Approximation
Designed for Molecules and Solids. Phys. Rev.
Lett..

[103] Vosko S. H., Wilk L., Nusair M. (1980). Accurate Spin-Dependent
Electron Liquid Correlation Energies for Local Spin Density Calculations:
A Critical Analysis. Can. J. Phys..

[104] Lee C., Yang W., Parr R. G. (1988). Development
of the Colle-Salvetti
Correlation-Energy Formula into a Functional of the Electron Density. Phys. Rev. B.

[105] Becke A. D. A. D. (1993). A New Mixing of Hartree-Fock and Local Density-Functional
Theories. J. Chem. Phys..

[106] Becke A. D. (1988). Density-Functional Exchange-Energy
Approximation with
Correct Asymptotic Behavior. Phys. Rev. A.

[107] Stephens P. J., Devlin F. J., Chabalowski C. F., Frisch M. J. (1994). Ab Initio Calculation
of Vibrational Absorption and
Circular Dichroism Spectra Using Density Functional Force Fields. J. Phys. Chem..

[108] Krishnan R., Binkley J. S., Seeger R., Pople J. A. (1980). Self-consistent
Molecular Orbital Methods. XX. A Basis Set for Correlated Wave Functions. J. Chem. Phys..

[109] Clark T., Chandrasekhar J., Spitznagel G. W., Schleyer P. V. R. (1983). Efficient Diffuse Function-augmented
Basis Sets for
Anion Calculations. III. The 3–21+ G Basis Set for First-row
Elements, Li–F. J. Comput. Chem..

[110] McLean A. D., Chandler G. S. (1980). Contracted Gaussian Basis Sets for
Molecular Calculations. I. Second Row Atoms, Z= 11–18. J. Chem. Phys..

[111] Francl M. M., Pietro W. J., Hehre W. J., Binkley J. S., Gordon M. S., DeFrees D. J., Pople J. A. (1982). Self-consistent
Molecular Orbital Methods. XXIII. A Polarization-type Basis Set for
Second-row Elements. J. Chem. Phys..

[112] Spitznagel G. W., Clark T., von Ragué Schleyer P., Hehre W. J. (1987). An Evaluation of
the Performance of Diffuse Function-augmented
Basis Sets for Second Row Elements, Na-Cl. J.
Comput. Chem..

[113] Frisch, M. J. ; Trucks, G. W. ; Schlegel, H. B. ; Scuseria, G. E. ; Robb, M. A. ; Cheeseman, J. R. ; Scalmani, G. ; Barone, V. ; Petersson, G. A. ; Nakatsuji, H. ; . Gaussian 16 Rev. C.01. Gaussian Inc.: Wallingford CT, 2016.

[114] Hirshfeld F. L. (1977). Bonded-Atom Fragments for Describing
Molecular Charge
Densities. Theor. Chim. Acta.

[115] Mehta N., Martin J. M. L. (2024). On the Sensitivity
of Computed Partial
Charges toward Basis Set and (Exchange-)­Correlation Treatment. J. Comput. Chem..

[116] Gubler M., Schäfer M. R., Behler J., Goedecker S. (2025). Accuracy of
Charge Densities in Electronic Structure Calculations. J. Chem. Phys..

[117] Heidar-Zadeh F., Ayers P. W., Verstraelen T., Vinogradov I., Vöhringer-Martinez E., Bultinck P. (2018). Information-Theoretic
Approaches to Atoms-in-Molecules: Hirshfeld Family of Partitioning
Schemes. J. Phys. Chem. A.

[118] Moltved K. A., Kepp K. P. (2020). Using Electronegativity
and Hardness
to Test Density Functionals. J. Chem. Phys..

[119] Barrera Y., Anderson J. S. M. (2022). Predicting the Reactivity of Unsaturated
Molecules to Methyl Radical Addition Using a Radical Two-Parameter
General-Purpose Reactivity Indicator. Chem.
Phys. Lett..

[120] Barrera Y., Anderson J. S. M. (2025). Comparative Study
of Predicting Radical
C-H Functionalization Sites in Nitrogen Heteroarenes Using a Radical
General-Purpose Reactivity Indicator and the Radical Fukui Function. J. Comput. Chem..

[121] Angelici R. J., Allison J. W. (1971). Stability Constants for Amino Acid
Coordination by Substituted Diethylenetriamine Complexes of Copper
(II) and the Kinetics of Amino Acid Ester Hydrolysis. Inorg. Chem..

[122] Barr D., Clegg W., Cowton L., Horsburgh L., Mackenzie F. M., Mulvey R. E. (1995). Lithium Anilide
Complexed by Pmdeta:
Expectation of a Simple Monomer, but in Reality an Odd Trinuclear
Composition Combining Three-, Four-and Five-Coordinate Lithium. J. Chem. Soc..

[123] Nanda A. K., Matyjaszewski K. (2003). Effect of [PMDETA]/[Cu (I)] Ratio,
Monomer, Solvent, Counterion, Ligand, and Alkyl Bromide on the Activation
Rate Constants in Atom Transfer Radical Polymerization. Macromolecules.

[124] Strohmann C., Gessner V. H. (2007). From the Alkyllithium
Aggregate [{(NBuLi)
2· PMDTA} 2] to Lithiated PMDTA. Angew.
Chem., Int. Ed..

[125] Jeftic L., Adams R. N. (1970). Electrochemical
Oxidation Pathways
of Benzo­[a]­Pyrene. J. Am. Chem. Soc..

[126] Johnson M. D., Calvin M. (1973). Induced Nucleophilic Substitution
in Benzo­[a]­Pyrene. Nature.

[127] Cavalieri E., Calvin M. (1971). Molecular Characteristics
of Some
Carcinogenic Hydrocarbons. Proc. Natl. Acad.
Sci. U. S. A..

[128] Bormann N., Ward J. S., Bergmann A. K., Wenz P., Rissanen K., Gong Y., Hatz W., Burbaum A., Mulks F. F. (2023). Diiminium Nucleophile Adducts Are Stable and Convenient
Strong Lewis Acids. Chem. - Eur. J..

[129] Maas G., Feith B. (1985). Azahexamethineneutrocyanines from
a N-(Tetramethylformamidinio) Pyridinium Salt. Angew. Chem., Int. Ed..

[130] Gong Y., Langwald J., Mulks F. F. (2024). On the
Road to Isolable
Geminal Carbodications. Chem.

[131] Kastrati A., Kremsmair A., Sunagatullina A. S., Korotenko V., Guersoy Y. C., Rout S. K., Lima F., Brocklehurst C. E., Karaghiosoff K., Zipse H., Knochel P. (2023). Calculation-Assisted
Regioselective Functionalization of the Imidazo­[1,2-a]­Pyrazine Scaffold
via Zinc and Magnesium Organometallic Intermediates. Chem. Sci..

[132] Stuyver T., Danovich D., De Proft F., Shaik S. (2019). Electrophilic
Aromatic Substitution Reactions: Mechanistic Landscape, Electrostatic
and Electric-Field Control of Reaction Rates, and Mechanistic Crossovers. J. Am. Chem. Soc..

[133] Mayr H., Bug T., Gotta M. F., Hering N., Irrgang B., Janker B., Kempf B., Loos R., Ofial A. R., Remennikov R., Schimmel H. (2001). Reference Scales for
the Characterization of Cationic Electrophiles and Neutral Nucleophiles. J. Am. Chem. Soc..

[134] Mayr H., Ofial A. R. (2008). Do General Nucleophilicity
Scales
Exist?. J. Phys. Org. Chem..

[135] Martínez-Araya J. I. (2013). Explaining Reaction
Mechanisms Using
the Dual Descriptor: A Complementary Tool to the Molecular Electrostatic
Potential. J. Mol. Model..

[136] Pearson R. G. (1987). Recent Advances in the Concept of
Hard and Soft Acids
and Bases. J. Chem. Edu..

[137] Leyssens T., Geerlings P., Peeters D. (2005). The Importance of the
External Potential on Group Electronegativity. J. Phys. Chem. A.

[138] Koch E. C. (2005). Acid-Base Interactions in Energetic
Materials: I. The
Hard and Soft Acids and Bases (HSAB) Principle–Insights to
Reactivity and Sensitivity of Energetic Materials. Propellants, Explos. Pyrotech. An Int. J. Deal. With Sci.
Technol. Asp. Energy Mater..

