# Supplementary material for: The Grand Canonical General-Purpose Reactivity Indicator: A Conceptual DFT Approach to Predict Molecular Reactivity and Experimental Electrophilicity and Nucleophilicity Scales
Source: J Chem Theory Comput. 2025 Oct 2;21(24):12508–22. doi: 10.1021/acs.jctc.5c00849 (PMC12746416; doi:10.1021/acs.jctc.5c00849)
Supplement: Supplementary file 1 [file ct5c00849_si_001.pdf]

Supporting Information (SI)

The Grand Canonical General-Purpose Reactivity Indicator: A Conceptual DFT Approach to Predict Molecular Reactivity and Experimental Electrophilicity and Nucleophilicity Scales

Yoshio Barrera,[1,2] Tomás Rocha-Rinza,[1] Florian F. Mulks,[2] Paul W. Ayers,[3] James S. M. Anderson[1*]

[1]Instituto de Química, Universidad Nacional Autónoma de México, Circuito Exterior, Ciudad Universitaria, Delegación Coyoacán, Ciudad de México, C.P. 04510, México

[2]Institute of Organic Chemistry, RWTH Aachen University, Landoltweg 1, 52074 Aachen, Germany

[3]Department of Chemistry and Chemical Biology, McMaster University, Hamilton L8S 4M1, Ontario, Canada

*Corresponding author: James S. M. Anderson,

e-mail: james.anderson@iquimica.unam.mx

Cases Studies (Benchmark)

Elucidation of Reactive Sites in Molecules with Multiple Susceptible Atoms.

Table S1. First choice of **CS1** (Scheme 1 in the main text), evaluated with the GC-GPRI for nucleophiles, Equation (22), $\Theta^{\kappa}_{\Delta\mu\leq 0,\alpha} = (\kappa+1)q^{(0)}_{Nu,\alpha} - \Delta\mu(\kappa-1)s^{(-)}_{Nu,\alpha}$. The functionals employed are: (**a**) ωB97XD, (**b**) M062X, (**c**) PBE0, (**d**) PBE, (**e**) TPSS and (**f**) SVWN, using the Hirshfeld population scheme. Cell colors indicate the atom with the lowest GC-GPRI values for each $\Delta\mu$ and κ. Values are reported in atomic units.

(**a**) **First Choice:** Atoms with the lowest GC-GPRI values at each $\Delta\mu$ and κ.

$\Delta\mu/\kappa$	1	0.8	0.6	0.4	0.2	0	-0.2	-0.4	-0.6	-0.8	-1
-1	-0.215	-0.314	-0.424	-0.534	-0.645	-0.755	-0.865	-0.976	-1.086	-1.196	-1.307
-0.8	-0.215	-0.288	-0.372	-0.456	-0.540	-0.624	-0.709	-0.793	-0.877	-0.961	-1.045
-0.6	-0.215	-0.261	-0.319	-0.378	-0.436	-0.494	-0.552	-0.610	-0.668	-0.726	-0.784
-0.4	-0.215	-0.235	-0.267	-0.299	-0.331	-0.363	-0.395	-0.427	-0.459	-0.491	-0.523
-0.2	-0.215	-0.209	-0.215	-0.221	-0.227	-0.232	-0.238	-0.244	-0.250	-0.256	-0.261
0.0	-0.215	-0.193	-0.172	-0.150	-0.129	-0.107	-0.086	-0.064	-0.043	-0.021	
N1						N2					

(**b**) **First Choice:** Atoms with the lowest GC-GPRI values at each $\Delta\mu$ and κ.

$\Delta\mu/\kappa$	1	0.8	0.6	0.4	0.2	0	-0.2	-0.4	-0.6	-0.8	-1
-1	-0.219	-0.311	-0.415	-0.519	-0.623	-0.727	-0.831	-0.935	-1.039	-1.143	-1.247
-0.8	-0.219	-0.286	-0.365	-0.444	-0.523	-0.602	-0.681	-0.761	-0.840	-0.919	-0.998
-0.6	-0.219	-0.261	-0.315	-0.369	-0.423	-0.478	-0.532	-0.586	-0.640	-0.694	-0.748
-0.4	-0.219	-0.236	-0.265	-0.294	-0.324	-0.353	-0.382	-0.411	-0.441	-0.470	-0.499
-0.2	-0.219	-0.211	-0.215	-0.219	-0.224	-0.228	-0.232	-0.237	-0.241	-0.245	-0.249
0.0	-0.219	-0.197	-0.175	-0.153	-0.131	-0.110	-0.088	-0.066	-0.044	-0.022	
N1						N2					

(**c**) **First Choice:** Atoms with the lowest GC-GPRI values at each $\Delta\mu$ and κ.

$\Delta\mu/\kappa$	1	0.8	0.6	0.4	0.2	0	-0.2	-0.4	-0.6	-0.8	-1
-1	-0.201	-0.247	-0.303	-0.360	-0.417	-0.474	-0.531	-0.587	-0.644	-0.701	-0.758
-0.8	-0.201	-0.231	-0.273	-0.315	-0.356	-0.398	-0.440	-0.481	-0.523	-0.565	-0.606
-0.6	-0.201	-0.216	-0.243	-0.269	-0.296	-0.322	-0.349	-0.375	-0.402	-0.428	-0.455
-0.4	-0.201	-0.203	-0.212	-0.224	-0.235	-0.246	-0.258	-0.269	-0.280	-0.292	-0.303
-0.2	-0.201	-0.191	-0.183	-0.178	-0.174	-0.171	-0.167	-0.163	-0.159	-0.155	-0.152
0.0	-0.201	-0.180	-0.160	-0.140	-0.120	-0.100	-0.080	-0.060	-0.040	-0.020	
N1						N2					

(**d**) **First Choice:** Atoms with the lowest GC-GPRI values at each $\Delta\mu$ and κ.

$\Delta\mu/\kappa$	1	0.8	0.6	0.4	0.2	0	-0.2	-0.4	-0.6	-0.8	-1
-1	-0.172	-0.221	-0.271	-0.322	-0.372	-0.422	-0.473	-0.523	-0.574	-0.624	-0.674
-0.8	-0.172	-0.208	-0.244	-0.281	-0.318	-0.355	-0.392	-0.429	-0.466	-0.503	-0.539
-0.6	-0.172	-0.194	-0.217	-0.241	-0.264	-0.288	-0.311	-0.334	-0.358	-0.381	-0.405
-0.4	-0.172	-0.181	-0.190	-0.200	-0.210	-0.220	-0.230	-0.240	-0.250	-0.260	-0.270
-0.2	-0.172	-0.168	-0.164	-0.160	-0.156	-0.153	-0.149	-0.146	-0.142	-0.138	-0.135
0.0	-0.172	-0.155	-0.138	-0.121	-0.103	-0.086	-0.069	-0.052	-0.034	-0.017	
N1						N2					

(e) **First Choice:** Atoms with the lowest GC-GPRI values at each $\Delta\mu$ and κ.

$\Delta\mu/\kappa$	1	0.8	0.6	0.4	0.2	0	-0.2	-0.4	-0.6	-0.8	-1
-1	-0.193	-0.240	-0.288	-0.336	-0.384	-0.432	-0.480	-0.528	-0.576	-0.624	-0.672
-0.8	-0.193	-0.226	-0.261	-0.295	-0.330	-0.364	-0.399	-0.434	-0.468	-0.503	-0.537
-0.6	-0.193	-0.213	-0.234	-0.255	-0.276	-0.297	-0.318	-0.340	-0.361	-0.382	-0.403
-0.4	-0.193	-0.200	-0.207	-0.215	-0.222	-0.230	-0.238	-0.246	-0.253	-0.261	-0.269
-0.2	-0.193	-0.187	-0.180	-0.174	-0.169	-0.163	-0.157	-0.152	-0.146	-0.140	-0.134
0.0	-0.193	-0.174	-0.155	-0.135	-0.116	-0.097	-0.077	-0.058	-0.039	-0.019	
N1						N2					

(f) **First Choice:** Atoms with the lowest GC-GPRI values at each $\Delta\mu$ and κ.

$\Delta\mu/\kappa$	1	0.8	0.6	0.4	0.2	0	-0.2	-0.4	-0.6	-0.8	-1
-1	-0.178	-0.223	-0.271	-0.319	-0.367	-0.415	-0.463	-0.511	-0.559	-0.607	-0.655
-0.8	-0.178	-0.211	-0.245	-0.280	-0.315	-0.350	-0.384	-0.419	-0.454	-0.489	-0.524
-0.6	-0.178	-0.198	-0.219	-0.241	-0.262	-0.284	-0.306	-0.328	-0.349	-0.371	-0.393
-0.4	-0.178	-0.185	-0.193	-0.201	-0.210	-0.219	-0.227	-0.236	-0.245	-0.253	-0.262
-0.2	-0.178	-0.173	-0.168	-0.163	-0.157	-0.153	-0.149	-0.144	-0.140	-0.135	-0.131
0.0	-0.178	-0.160	-0.142	-0.124	-0.107	-0.089	-0.071	-0.053	-0.036	-0.018	
N1						N2					

Table S2. Reactivity transition tables of **CS2** (Scheme 2 in the main text), evaluated with the GC-GPRI for nucleophiles, Equation (22), $\Theta^{\kappa}_{\Delta\mu\leq0,\alpha} = (\kappa+1)q^{(0)}_{Nu,\alpha} - \Delta\mu(\kappa-1)s^{(-)}_{Nu,\alpha}$. The functionals employed are: (**a**) ωB97XD, (**b**) M062X, (**c**) PBE0, (**d**) PBE, (**e**) TPSS and (**f**) SVWN, using the Hirshfeld population scheme. First (**1**) and second (**2**) choices show the most and second most reactive atoms, respectively. Cell colors indicate the atom with the lowest GC-GPRI values for each $\Delta\mu$ and κ. Values are reported in atomic units.

(**a.1**) **First Choice:** Atoms with the lowest GC-GPRI values at each $\Delta\mu$ and κ.

$\Delta\mu/\kappa$	1	0.8	0.6	0.4	0.2	0	-0.2	-0.4	-0.6	-0.8	-1
-1	-0.088	-0.146	-0.208	-0.269	-0.331	-0.392	-0.454	-0.516	-0.577	-0.639	-0.700
-0.8	-0.088	-0.132	-0.180	-0.227	-0.275	-0.322	-0.370	-0.418	-0.465	-0.513	-0.560
-0.6	-0.088	-0.118	-0.152	-0.185	-0.219	-0.252	-0.286	-0.319	-0.353	-0.387	-0.420
-0.4	-0.088	-0.104	-0.124	-0.143	-0.163	-0.182	-0.202	-0.221	-0.241	-0.261	-0.280
-0.2	-0.088	-0.090	-0.096	-0.101	-0.107	-0.112	-0.118	-0.123	-0.129	-0.135	-0.140
0.0	-0.088	-0.079	-0.070	-0.062	-0.053	-0.044	-0.035	-0.026	-0.018	-0.009	

C1 C2 C3 C6 C8

(**a.2**) **Second Choice:** Atoms with the second lowest GC-GPRI values at each $\Delta\mu$ and κ.

$\Delta\mu/\kappa$	1	0.8	0.6	0.4	0.2	0	-0.2	-0.4	-0.6	-0.8	-1
-1	-0.088	-0.133	-0.180	-0.227	-0.274	-0.321	-0.368	-0.415	-0.462	-0.509	-0.556
-0.8	-0.088	-0.122	-0.157	-0.193	-0.229	-0.265	-0.301	-0.337	-0.373	-0.409	-0.444
-0.6	-0.088	-0.110	-0.135	-0.160	-0.185	-0.209	-0.234	-0.259	-0.284	-0.309	-0.333
-0.4	-0.088	-0.099	-0.113	-0.127	-0.140	-0.154	-0.168	-0.181	-0.195	-0.209	-0.222
-0.2	-0.088	-0.089	-0.091	-0.093	-0.096	-0.098	-0.101	-0.103	-0.106	-0.109	-0.111
0.0	-0.088	-0.079	-0.070	-0.061	-0.053	-0.044	-0.035	-0.026	-0.018	-0.009	

C1 C2 C3 C6 C8

(**b.1**) **First Choice:** Atoms with the lowest GC-GPRI values at each $\Delta\mu$ and κ.

$\Delta\mu/\kappa$	1	0.8	0.6	0.4	0.2	0	-0.2	-0.4	-0.6	-0.8	-1
-1	-0.086	-0.142	-0.203	-0.263	-0.324	-0.385	-0.446	-0.507	-0.568	-0.629	-0.689
-0.8	-0.086	-0.128	-0.175	-0.222	-0.269	-0.316	-0.363	-0.410	-0.457	-0.504	-0.552
-0.6	-0.086	-0.114	-0.147	-0.181	-0.214	-0.247	-0.281	-0.314	-0.347	-0.380	-0.414
-0.4	-0.086	-0.100	-0.120	-0.139	-0.159	-0.178	-0.198	-0.217	-0.237	-0.256	-0.276
-0.2	-0.086	-0.087	-0.092	-0.098	-0.104	-0.109	-0.115	-0.121	-0.126	-0.132	-0.138
0.0	-0.086	-0.078	-0.069	-0.060	-0.052	-0.043	-0.034	-0.026	-0.017	-0.009	

C1 C2 C3 C6 C8

(**b.2**) **Second Choice:** Atoms with the second lowest GC-GPRI values at each $\Delta\mu$ and κ.

$\Delta\mu/\kappa$	1	0.8	0.6	0.4	0.2	0	-0.2	-0.4	-0.6	-0.8	-1
-1	-0.086	-0.129	-0.176	-0.223	-0.270	-0.317	-0.363	-0.410	-0.457	-0.504	-0.551
-0.8	-0.086	-0.118	-0.154	-0.190	-0.226	-0.262	-0.297	-0.333	-0.369	-0.405	-0.441
-0.6	-0.086	-0.107	-0.132	-0.157	-0.182	-0.206	-0.231	-0.256	-0.281	-0.306	-0.330
-0.4	-0.086	-0.096	-0.110	-0.124	-0.138	-0.151	-0.165	-0.179	-0.193	-0.207	-0.220
-0.2	-0.086	-0.086	-0.088	-0.091	-0.094	-0.096	-0.099	-0.102	-0.105	-0.107	-0.110
0.0	-0.086	-0.077	-0.069	-0.060	-0.052	-0.043	-0.034	-0.026	-0.017	-0.009	

C1 C2 C3 C6 C8

(**c.1**) **First Choice:** Atoms with the lowest GC-GPRI values at each $\Delta\mu$ and κ.

$\Delta\mu/\kappa$	1	0.8	0.6	0.4	0.2	0	-0.2	-0.4	-0.6	-0.8	-1
-1	-0.090	-0.142	-0.199	-0.256	-0.313	-0.370	-0.427	-0.484	-0.541	-0.598	-0.655
-0.8	-0.090	-0.129	-0.173	-0.217	-0.261	-0.305	-0.349	-0.393	-0.437	-0.480	-0.524
-0.6	-0.090	-0.116	-0.147	-0.178	-0.208	-0.239	-0.270	-0.301	-0.332	-0.362	-0.393
-0.4	-0.090	-0.103	-0.121	-0.138	-0.156	-0.174	-0.191	-0.209	-0.227	-0.244	-0.262
-0.2	-0.090	-0.090	-0.094	-0.099	-0.104	-0.108	-0.113	-0.117	-0.122	-0.127	-0.131
0.0	-0.090	-0.081	-0.072	-0.063	-0.054	-0.045	-0.036	-0.027	-0.018	-0.009	

C1 C2 C3 C6 C8

(**c.2**) **Second Choice:** Atoms with the second lowest GC-GPRI values at each $\Delta\mu$ and κ.

$\Delta\mu/\kappa$	1	0.8	0.6	0.4	0.2	0	-0.2	-0.4	-0.6	-0.8	-1
-1	-0.090	-0.133	-0.179	-0.225	-0.272	-0.318	-0.364	-0.411	-0.457	-0.503	-0.550
-0.8	-0.090	-0.122	-0.157	-0.192	-0.228	-0.263	-0.298	-0.334	-0.369	-0.404	-0.440
-0.6	-0.090	-0.111	-0.135	-0.159	-0.184	-0.208	-0.232	-0.257	-0.281	-0.305	-0.330
-0.4	-0.090	-0.100	-0.113	-0.127	-0.140	-0.153	-0.167	-0.180	-0.193	-0.207	-0.220
-0.2	-0.090	-0.089	-0.091	-0.094	-0.096	-0.098	-0.101	-0.103	-0.105	-0.108	-0.110
0.0	-0.090	-0.081	-0.072	-0.063	-0.054	-0.045	-0.036	-0.027	-0.018	-0.009	

C1 C2 C3 C6 C8

(**d.1**) **First Choice:** Atoms with the lowest GC-GPRI values at each $\Delta\mu$ and κ.

$\Delta\mu/\kappa$	1	0.8	0.6	0.4	0.2	0	-0.2	-0.4	-0.6	-0.8	-1
-1	-0.088	-0.135	-0.187	-0.239	-0.291	-0.343	-0.396	-0.448	-0.500	-0.552	-0.604
-0.8	-0.088	-0.123	-0.163	-0.203	-0.243	-0.283	-0.323	-0.363	-0.403	-0.443	-0.483
-0.6	-0.088	-0.111	-0.139	-0.167	-0.195	-0.223	-0.251	-0.279	-0.307	-0.334	-0.362
-0.4	-0.088	-0.099	-0.115	-0.131	-0.146	-0.162	-0.178	-0.194	-0.210	-0.226	-0.242
-0.2	-0.088	-0.087	-0.091	-0.094	-0.098	-0.102	-0.106	-0.109	-0.113	-0.117	-0.121
0.0	-0.088	-0.079	-0.070	-0.062	-0.053	-0.044	-0.035	-0.026	-0.018	-0.009	

C1 C2 C6 C8 C9

(**d.2**) **Second Choice:** Atoms with the second lowest GC-GPRI values at each $\Delta\mu$ and κ.

$\Delta\mu/\kappa$	1	0.8	0.6	0.4	0.2	0	-0.2	-0.4	-0.6	-0.8	-1
-1	-0.088	-0.129	-0.174	-0.220	-0.265	-0.310	-0.355	-0.400	-0.445	-0.490	-0.536
-0.8	-0.088	-0.119	-0.153	-0.187	-0.222	-0.256	-0.291	-0.325	-0.360	-0.394	-0.428
-0.6	-0.088	-0.108	-0.132	-0.155	-0.179	-0.203	-0.226	-0.250	-0.274	-0.298	-0.321
-0.4	-0.088	-0.097	-0.110	-0.123	-0.136	-0.149	-0.162	-0.175	-0.188	-0.201	-0.214
-0.2	-0.088	-0.087	-0.089	-0.091	-0.093	-0.096	-0.098	-0.100	-0.103	-0.105	-0.107
0.0	-0.088	-0.079	-0.070	-0.061	-0.053	-0.044	-0.035	-0.026	-0.018	-0.009	

C1 C2 C6 C8 C9

(**e.1**) **First Choice:** Atoms with the lowest GC-GPRI values at each $\Delta\mu$ and κ.

$\Delta\mu/\kappa$	1	0.8	0.6	0.4	0.2	0	-0.2	-0.4	-0.6	-0.8	-1
-1	-0.080	-0.129	-0.183	-0.237	-0.291	-0.345	-0.398	-0.452	-0.506	-0.560	-0.614
-0.8	-0.080	-0.117	-0.159	-0.200	-0.242	-0.283	-0.325	-0.366	-0.408	-0.449	-0.491
-0.6	-0.080	-0.105	-0.134	-0.163	-0.193	-0.222	-0.251	-0.280	-0.310	-0.339	-0.368
-0.4	-0.080	-0.093	-0.110	-0.127	-0.144	-0.161	-0.178	-0.194	-0.211	-0.228	-0.245
-0.2	-0.080	-0.080	-0.085	-0.090	-0.094	-0.099	-0.104	-0.109	-0.113	-0.118	-0.123
0.0	-0.080	-0.072	-0.064	-0.056	-0.048	-0.040	-0.032	-0.024	-0.016	-0.008	

C1 C2 C6 C8 C9

(**e.2**) **Second Choice:** Atoms with the second lowest GC-GPRI values at each $\Delta\mu$ and κ.

$\Delta\mu/\kappa$	1	0.8	0.6	0.4	0.2	0	-0.2	-0.4	-0.6	-0.8	-1
-1	-0.080	-0.123	-0.169	-0.215	-0.262	-0.308	-0.354	-0.400	-0.447	-0.493	-0.539
-0.8	-0.080	-0.112	-0.148	-0.183	-0.219	-0.254	-0.289	-0.325	-0.360	-0.396	-0.431
-0.6	-0.080	-0.101	-0.126	-0.151	-0.175	-0.200	-0.225	-0.249	-0.274	-0.299	-0.323
-0.4	-0.080	-0.091	-0.104	-0.118	-0.132	-0.146	-0.160	-0.174	-0.188	-0.202	-0.216
-0.2	-0.080	-0.080	-0.083	-0.086	-0.089	-0.092	-0.095	-0.098	-0.102	-0.105	-0.108
0.0	-0.080	-0.072	-0.064	-0.056	-0.048	-0.040	-0.032	-0.024	-0.016	-0.008	

C1 C2 C6 C8 C9

(f.1) First Choice: Atoms with the lowest GC-GPRI values at each $\Delta\mu$ and κ.

$\Delta\mu/\kappa$	1	0.8	0.6	0.4	0.2	0	-0.2	-0.4	-0.6	-0.8	-1
-1	-0.101	-0.144	-0.193	-0.243	-0.293	-0.342	-0.392	-0.442	-0.491	-0.541	-0.591
-0.8	-0.101	-0.132	-0.170	-0.208	-0.245	-0.283	-0.321	-0.359	-0.397	-0.435	-0.473
-0.6	-0.101	-0.120	-0.146	-0.172	-0.198	-0.224	-0.250	-0.276	-0.302	-0.328	-0.354
-0.4	-0.101	-0.108	-0.122	-0.137	-0.151	-0.165	-0.179	-0.194	-0.208	-0.222	-0.236
-0.2	-0.101	-0.098	-0.099	-0.101	-0.104	-0.106	-0.108	-0.111	-0.113	-0.116	-0.118
0.0	-0.101	-0.091	-0.081	-0.071	-0.061	-0.050	-0.040	-0.030	-0.020	-0.010	

C1	C2	C6	C8	C9

(f.2) Second Choice: Atoms with the second lowest GC-GPRI values at each $\Delta\mu$ and κ.

$\Delta\mu/\kappa$	1	0.8	0.6	0.4	0.2	0	-0.2	-0.4	-0.6	-0.8	-1
-1	-0.101	-0.139	-0.183	-0.226	-0.269	-0.312	-0.356	-0.399	-0.442	-0.485	-0.529
-0.8	-0.101	-0.129	-0.162	-0.194	-0.227	-0.260	-0.292	-0.325	-0.358	-0.390	-0.423
-0.6	-0.101	-0.118	-0.140	-0.162	-0.185	-0.207	-0.229	-0.251	-0.273	-0.295	-0.317
-0.4	-0.101	-0.108	-0.119	-0.131	-0.142	-0.154	-0.165	-0.177	-0.188	-0.200	-0.211
-0.2	-0.101	-0.098	-0.098	-0.099	-0.100	-0.101	-0.102	-0.103	-0.104	-0.105	-0.106
0.0	-0.101	-0.091	-0.081	-0.071	-0.060	-0.050	-0.040	-0.030	-0.020	-0.010	

C1	C2	C6	C8	C9

Table S3. Reactivity transition tables for **CS3** (Scheme 3 in the main text), evaluated with the GC-GPRI for electrophiles, Equation (29), $\Theta^{\kappa}_{\Delta\mu\geq 0,\beta} = -(\kappa+1)q^{(0)}_{Ele,\beta} + \Delta\mu(\kappa-1)s^{(+)}_{Ele,\beta}$. The functionals employed are: (**a**) ωB97XD, (**b**) M062X, (**c**) PBE0, (**d**) PBE, (**e**) TPSS and (**f**) SVWN, using the Hirshfeld population scheme. First (**1**) and second (**2**) choices show the most and second most reactive atoms, respectively. Cell colors indicate the atom with the lowest GC-GPRI values for each $\Delta\mu$ and κ. Values are reported in atomic units.

(**a.1**) **First Choice:** Atoms with the lowest GC-GPRI values at each $\Delta\mu$ and κ.

$\Delta\mu/\kappa$	1	0.8	0.6	0.4	0.2	0	-0.2	-0.4	-0.6	-0.8	-1
0	-0.416	-0.374	-0.333	-0.291	-0.249	-0.208	-0.166	-0.125	-0.083	-0.042	
0.2	-0.416	-0.377	-0.339	-0.301	-0.263	-0.224	-0.186	-0.171	-0.171	-0.171	-0.172
0.4	-0.416	-0.381	-0.346	-0.311	-0.276	-0.256	-0.273	-0.291	-0.309	-0.326	-0.344
0.6	-0.416	-0.384	-0.352	-0.320	-0.307	-0.342	-0.376	-0.411	-0.446	-0.481	-0.516
0.8	-0.416	-0.387	-0.359	-0.330	-0.376	-0.428	-0.480	-0.532	-0.584	-0.636	-0.688
1	-0.416	-0.391	-0.365	-0.375	-0.444	-0.514	-0.583	-0.652	-0.721	-0.790	-0.859
C1			C2			C4			H[a]		

(**a.2**) **Second Choice:** Atoms with the second lowest GC-GPRI values at each $\Delta\mu$ and κ.

$\Delta\mu/\kappa$	1	0.8	0.6	0.4	0.2	0	-0.2	-0.4	-0.6	-0.8	-1
0	-0.226	-0.203	-0.181	-0.158	-0.136	-0.113	-0.090	-0.068	-0.045	-0.023	
0.2	-0.226	-0.211	-0.195	-0.180	-0.169	-0.170	-0.170	-0.148	-0.137	-0.132	-0.127
0.4	-0.226	-0.218	-0.210	-0.221	-0.238	-0.240	-0.223	-0.231	-0.239	-0.246	-0.254
0.6	-0.226	-0.225	-0.237	-0.272	-0.289	-0.279	-0.300	-0.320	-0.340	-0.361	-0.381
0.8	-0.226	-0.233	-0.272	-0.324	-0.310	-0.343	-0.376	-0.409	-0.442	-0.475	-0.508
1	-0.226	-0.240	-0.306	-0.340	-0.360	-0.406	-0.452	-0.498	-0.543	-0.589	-0.635
C1			C2			C4			H[a]		

(**b.1**) **First Choice:** Atoms with the lowest GC-GPRI values at each $\Delta\mu$ and κ.

$\Delta\mu/\kappa$	1	0.8	0.6	0.4	0.2	0	-0.2	-0.4	-0.6	-0.8	-1
0	-0.411	-0.370	-0.329	-0.288	-0.247	-0.206	-0.165	-0.123	-0.082	-0.041	
0.2	-0.411	-0.373	-0.336	-0.298	-0.260	-0.222	-0.184	-0.167	-0.167	-0.167	-0.167
0.4	-0.411	-0.377	-0.342	-0.308	-0.273	-0.250	-0.267	-0.284	-0.301	-0.317	-0.334
0.6	-0.411	-0.380	-0.349	-0.318	-0.300	-0.334	-0.367	-0.401	-0.434	-0.468	-0.501
0.8	-0.411	-0.383	-0.356	-0.328	-0.367	-0.417	-0.468	-0.518	-0.568	-0.618	-0.668
1	-0.411	-0.387	-0.362	-0.367	-0.434	-0.501	-0.568	-0.635	-0.701	-0.768	-0.835
C1			C2			C4			H[a]		

(**b.2**) **Second Choice:** Atoms with the second lowest GC-GPRI values at each $\Delta\mu$ and κ.

$\Delta\mu/\kappa$	1	0.8	0.6	0.4	0.2	0	-0.2	-0.4	-0.6	-0.8	-1
0	-0.225	-0.202	-0.180	-0.157	-0.135	-0.112	-0.090	-0.067	-0.045	-0.022	
0.2	-0.225	-0.209	-0.194	-0.179	-0.167	-0.167	-0.167	-0.147	-0.134	-0.128	-0.123
0.4	-0.225	-0.216	-0.208	-0.217	-0.234	-0.239	-0.218	-0.225	-0.232	-0.238	-0.245
0.6	-0.225	-0.224	-0.234	-0.267	-0.287	-0.273	-0.292	-0.311	-0.330	-0.349	-0.368
0.8	-0.225	-0.231	-0.267	-0.317	-0.303	-0.334	-0.365	-0.397	-0.428	-0.459	-0.490
1	-0.225	-0.238	-0.300	-0.338	-0.352	-0.395	-0.439	-0.482	-0.526	-0.569	-0.613
C1			C2			C4			H[a]		

(**c.1**) **First Choice:** Atoms with the lowest GC-GPRI values at each $\Delta\mu$ and κ.

$\Delta\mu/\kappa$	1	0.8	0.6	0.4	0.2	0	-0.2	-0.4	-0.6	-0.8	-1
0	-0.391	-0.352	-0.313	-0.274	-0.235	-0.196	-0.157	-0.117	-0.078	-0.039	
0.2	-0.391	-0.357	-0.322	-0.287	-0.252	-0.218	-0.183	-0.164	-0.164	-0.165	-0.165
0.4	-0.391	-0.361	-0.331	-0.300	-0.270	-0.246	-0.263	-0.280	-0.297	-0.314	-0.331
0.6	-0.391	-0.365	-0.339	-0.313	-0.295	-0.328	-0.362	-0.395	-0.429	-0.462	-0.496
0.8	-0.391	-0.370	-0.348	-0.326	-0.361	-0.411	-0.461	-0.511	-0.561	-0.611	-0.661
1	-0.391	-0.374	-0.357	-0.361	-0.427	-0.494	-0.560	-0.627	-0.693	-0.760	-0.826
C1			C2			C4			H[a]		

(**c.2**) **Second Choice:** Atoms with the second lowest GC-GPRI values at each $\Delta\mu$ and κ.

$\Delta\mu/\kappa$	1	0.8	0.6	0.4	0.2	0	-0.2	-0.4	-0.6	-0.8	-1
0	-0.226	-0.204	-0.181	-0.158	-0.136	-0.113	-0.091	-0.068	-0.045	-0.023	
0.2	-0.226	-0.211	-0.195	-0.180	-0.164	-0.163	-0.164	-0.148	-0.131	-0.126	-0.121
0.4	-0.226	-0.218	-0.210	-0.212	-0.229	-0.239	-0.213	-0.220	-0.227	-0.234	-0.241
0.6	-0.226	-0.225	-0.228	-0.261	-0.287	-0.266	-0.285	-0.304	-0.323	-0.343	-0.362
0.8	-0.226	-0.232	-0.261	-0.311	-0.305	-0.326	-0.357	-0.389	-0.420	-0.451	-0.482
1	-0.226	-0.240	-0.294	-0.340	-0.343	-0.387	-0.430	-0.473	-0.516	-0.560	-0.603
C1			C2			C4			H[a]		

(**d.1**) **First Choice:** Atoms with the lowest GC-GPRI values at each $\Delta\mu$ and κ.

$\Delta\mu/\kappa$	1	0.8	0.6	0.4	0.2	0	-0.2	-0.4	-0.6	-0.8	-1
0	-0.348	-0.313	-0.278	-0.244	-0.209	-0.174	-0.139	-0.104	-0.070	-0.035	
0.2	-0.348	-0.319	-0.289	-0.260	-0.231	-0.202	-0.172	-0.154	-0.154	-0.155	-0.155
0.4	-0.348	-0.324	-0.300	-0.277	-0.253	-0.231	-0.246	-0.262	-0.278	-0.294	-0.310
0.6	-0.348	-0.330	-0.311	-0.293	-0.277	-0.308	-0.340	-0.371	-0.403	-0.434	-0.465
0.8	-0.348	-0.335	-0.322	-0.310	-0.339	-0.386	-0.433	-0.480	-0.527	-0.574	-0.621
1	-0.348	-0.341	-0.333	-0.338	-0.401	-0.463	-0.526	-0.588	-0.651	-0.713	-0.776
C1			C2			C4			H[a]		

(**d.2**) **Second Choice:** Atoms with the second lowest GC-GPRI values at each $\Delta\mu$ and κ.

$\Delta\mu/\kappa$	1	0.8	0.6	0.4	0.2	0	-0.2	-0.4	-0.6	-0.8	-1
0	-0.222	-0.200	-0.178	-0.156	-0.133	-0.111	-0.089	-0.067	-0.044	-0.022	
0.2	-0.222	-0.207	-0.192	-0.177	-0.161	-0.153	-0.153	-0.143	-0.120	-0.115	-0.111
0.4	-0.222	-0.214	-0.206	-0.199	-0.215	-0.229	-0.205	-0.202	-0.209	-0.215	-0.222
0.6	-0.222	-0.221	-0.220	-0.245	-0.275	-0.257	-0.262	-0.280	-0.297	-0.315	-0.332
0.8	-0.222	-0.228	-0.245	-0.292	-0.297	-0.300	-0.329	-0.357	-0.386	-0.414	-0.443
1	-0.222	-0.235	-0.276	-0.326	-0.319	-0.356	-0.395	-0.435	-0.475	-0.514	-0.554
C1			C2			C4			H[a]		

(**e.1**) **First Choice:** Atoms with the lowest GC-GPRI values at each $\Delta\mu$ and κ.

$\Delta\mu/\kappa$	1	0.8	0.6	0.4	0.2	0	-0.2	-0.4	-0.6	-0.8	-1
0	-0.368	-0.331	-0.295	-0.258	-0.221	-0.184	-0.147	-0.110	-0.074	-0.037	
0.2	-0.368	-0.337	-0.305	-0.274	-0.243	-0.211	-0.180	-0.157	-0.157	-0.156	-0.156
0.4	-0.368	-0.342	-0.316	-0.290	-0.264	-0.239	-0.251	-0.266	-0.282	-0.297	-0.312
0.6	-0.368	-0.348	-0.327	-0.307	-0.286	-0.314	-0.345	-0.376	-0.406	-0.437	-0.468
0.8	-0.368	-0.353	-0.338	-0.323	-0.346	-0.392	-0.438	-0.485	-0.531	-0.577	-0.624
1	-0.368	-0.359	-0.349	-0.346	-0.408	-0.470	-0.532	-0.594	-0.656	-0.718	-0.780
C1			C2			C4			H[a]		

(**e.2**) **Second Choice:** Atoms with the second lowest GC-GPRI values at each $\Delta\mu$ and κ.

$\Delta\mu/\kappa$	1	0.8	0.6	0.4	0.2	0	-0.2	-0.4	-0.6	-0.8	-1
0	-0.216	-0.194	-0.173	-0.151	-0.130	-0.108	-0.086	-0.065	-0.043	-0.022	
0.2	-0.216	-0.201	-0.187	-0.172	-0.159	-0.158	-0.158	-0.149	-0.123	-0.118	-0.112
0.4	-0.216	-0.208	-0.201	-0.206	-0.221	-0.236	-0.213	-0.208	-0.213	-0.218	-0.223
0.6	-0.216	-0.215	-0.222	-0.253	-0.284	-0.266	-0.269	-0.286	-0.302	-0.319	-0.335
0.8	-0.216	-0.222	-0.253	-0.300	-0.308	-0.309	-0.336	-0.364	-0.392	-0.419	-0.447
1	-0.216	-0.229	-0.284	-0.339	-0.330	-0.365	-0.403	-0.442	-0.481	-0.520	-0.558
C1			C2			C4			H[a]		

(f.1) First Choice: Atoms with the lowest GC-GPRI values at each $\Delta\mu$ and κ.

$\Delta\mu/\kappa$	1	0.8	0.6	0.4	0.2	0	-0.2	-0.4	-0.6	-0.8	-1
0	-0.326	-0.294	-0.261	-0.228	-0.196	-0.163	-0.130	-0.098	-0.065	-0.033	
0.2	-0.326	-0.299	-0.272	-0.245	-0.218	-0.191	-0.164	-0.149	-0.151	-0.153	-0.155
0.4	-0.326	-0.305	-0.283	-0.261	-0.240	-0.223	-0.241	-0.258	-0.275	-0.293	-0.310
0.6	-0.326	-0.310	-0.294	-0.278	-0.268	-0.301	-0.334	-0.367	-0.399	-0.432	-0.465
0.8	-0.326	-0.316	-0.305	-0.295	-0.330	-0.378	-0.427	-0.475	-0.524	-0.572	-0.621
1	-0.326	-0.321	-0.316	-0.328	-0.392	-0.456	-0.520	-0.584	-0.648	-0.712	-0.776
C1			C2			C4			H^a		

(f.2) Second Choice: Atoms with the second lowest GC-GPRI values at each $\Delta\mu$ and κ.

$\Delta\mu/\kappa$	1	0.8	0.6	0.4	0.2	0	-0.2	-0.4	-0.6	-0.8	-1
0	-0.236	-0.213	-0.189	-0.165	-0.142	-0.118	-0.095	-0.071	-0.047	-0.024	
0.2	-0.236	-0.220	-0.203	-0.186	-0.170	-0.153	-0.147	-0.137	-0.116	-0.113	-0.110
0.4	-0.236	-0.227	-0.217	-0.207	-0.206	-0.218	-0.197	-0.196	-0.204	-0.212	-0.220
0.6	-0.236	-0.234	-0.231	-0.235	-0.262	-0.246	-0.254	-0.273	-0.292	-0.311	-0.330
0.8	-0.236	-0.240	-0.245	-0.281	-0.284	-0.290	-0.320	-0.350	-0.380	-0.410	-0.440
1	-0.236	-0.247	-0.264	-0.311	-0.306	-0.345	-0.386	-0.427	-0.468	-0.509	-0.550
C1			C2			C4			H^a		

Table S4. Reactivity transition tables for **CS4** (Scheme 4 in the main text), evaluated with the GC-GPRI for electrophiles, Equation (29), $\Theta^{\kappa}_{\Delta\mu\geq 0,\beta} = -(\kappa+1)q^{(0)}_{Ele,\beta} + \Delta\mu(\kappa-1)s^{(+)}_{Ele,\beta}$. The functionals employed are: (**a**) ωB97XD, (**b**) M062X, (**c**) PBE0, (**d**) PBE, (**e**) TPSS and (**f**) SVWN, using the Hirshfeld population scheme. First (**1**) and second (**2**) choices show the most and second most reactive atoms, respectively. Cell colors indicate the atom with the lowest GC-GPRI values for each $\Delta\mu$ and κ. Values are reported in atomic units.

(**a.1**) **First Choice:** Atoms with the lowest GC-GPRI values at each $\Delta\mu$ and κ.

$\Delta\mu/\kappa$	1	0.8	0.6	0.4	0.2	0	-0.2	-0.4	-0.6	-0.8	-1
0	-0.138	-0.124	-0.110	-0.097	-0.083	-0.069	-0.055	-0.041	-0.028	-0.014	
0.2	-0.138	-0.133	-0.128	-0.124	-0.134	-0.145	-0.155	-0.165	-0.175	-0.186	-0.196
0.4	-0.138	-0.142	-0.153	-0.183	-0.213	-0.243	-0.272	-0.302	-0.332	-0.362	-0.392
0.6	-0.138	-0.150	-0.192	-0.242	-0.291	-0.340	-0.390	-0.439	-0.489	-0.538	-0.588
0.8	-0.138	-0.162	-0.231	-0.300	-0.369	-0.438	-0.507	-0.576	-0.645	-0.714	-0.783
1	-0.138	-0.182	-0.271	-0.359	-0.448	-0.536	-0.625	-0.713	-0.802	-0.891	-0.979

C1	C4	C9	H^a	H^b

(**a.2**) **Second Choice:** Atoms with the second lowest GC-GPRI values at each $\Delta\mu$ and κ.

$\Delta\mu/\kappa$	1	0.8	0.6	0.4	0.2	0	-0.2	-0.4	-0.6	-0.8	-1
0	-0.136	-0.123	-0.109	-0.096	-0.082	-0.068	-0.055	-0.041	-0.027	-0.014	
0.2	-0.136	-0.131	-0.125	-0.123	-0.118	-0.113	-0.108	-0.103	-0.116	-0.131	-0.146
0.4	-0.136	-0.138	-0.145	-0.149	-0.153	-0.156	-0.175	-0.204	-0.233	-0.262	-0.291
0.6	-0.136	-0.146	-0.163	-0.175	-0.188	-0.218	-0.262	-0.306	-0.349	-0.393	-0.437
0.8	-0.136	-0.159	-0.180	-0.201	-0.233	-0.291	-0.349	-0.407	-0.466	-0.524	-0.582
1	-0.136	-0.168	-0.198	-0.228	-0.291	-0.364	-0.437	-0.509	-0.582	-0.655	-0.728

C1	C4	C9	H^a	H^b

(**b.1**) **First Choice:** Atoms with the lowest GC-GPRI values at each $\Delta\mu$ and κ.

$\Delta\mu/\kappa$	1	0.8	0.6	0.4	0.2	0	-0.2	-0.4	-0.6	-0.8	-1
0	-0.137	-0.123	-0.110	-0.096	-0.082	-0.068	-0.055	-0.041	-0.027	-0.014	
0.2	-0.137	-0.132	-0.127	-0.122	-0.132	-0.142	-0.151	-0.161	-0.171	-0.181	-0.191
0.4	-0.137	-0.140	-0.150	-0.179	-0.208	-0.237	-0.266	-0.295	-0.324	-0.354	-0.383
0.6	-0.137	-0.149	-0.188	-0.236	-0.285	-0.333	-0.381	-0.429	-0.478	-0.526	-0.574
0.8	-0.137	-0.159	-0.226	-0.294	-0.361	-0.429	-0.496	-0.563	-0.631	-0.698	-0.765
1	-0.137	-0.178	-0.265	-0.351	-0.438	-0.524	-0.611	-0.697	-0.784	-0.870	-0.957

C1	C4	C9	H^a	H^b

(**b.2**) **Second Choice:** Atoms with the second lowest GC-GPRI values at each $\Delta\mu$ and κ.

$\Delta\mu/\kappa$	1	0.8	0.6	0.4	0.2	0	-0.2	-0.4	-0.6	-0.8	-1
0	-0.136	-0.122	-0.108	-0.095	-0.081	-0.068	-0.054	-0.041	-0.027	-0.014	
0.2	-0.136	-0.130	-0.124	-0.122	-0.116	-0.111	-0.106	-0.102	-0.117	-0.131	-0.145
0.4	-0.136	-0.137	-0.144	-0.147	-0.151	-0.154	-0.175	-0.204	-0.232	-0.261	-0.290
0.6	-0.136	-0.145	-0.161	-0.173	-0.185	-0.219	-0.262	-0.305	-0.348	-0.392	-0.435
0.8	-0.136	-0.158	-0.178	-0.199	-0.234	-0.291	-0.349	-0.407	-0.464	-0.522	-0.580
1	-0.136	-0.166	-0.195	-0.224	-0.292	-0.364	-0.436	-0.508	-0.580	-0.652	-0.724

C1	C4	C9	H^a	H^b

(c.1) First Choice: Atoms with the lowest GC-GPRI values at each $\Delta\mu$ and κ.

$\Delta\mu/\kappa$	1	0.8	0.6	0.4	0.2	0	-0.2	-0.4	-0.6	-0.8	-1
0	-0.137	-0.123	-0.110	-0.096	-0.082	-0.069	-0.055	-0.041	-0.027	-0.014	
0.2	-0.137	-0.132	-0.127	-0.122	-0.124	-0.135	-0.146	-0.157	-0.168	-0.179	-0.190
0.4	-0.137	-0.141	-0.144	-0.170	-0.200	-0.230	-0.260	-0.289	-0.319	-0.349	-0.379
0.6	-0.137	-0.149	-0.178	-0.227	-0.276	-0.325	-0.373	-0.422	-0.471	-0.520	-0.568
0.8	-0.137	-0.158	-0.216	-0.284	-0.352	-0.419	-0.487	-0.555	-0.623	-0.690	-0.758
1	-0.137	-0.167	-0.254	-0.341	-0.427	-0.514	-0.601	-0.687	-0.774	-0.861	-0.947

C1 C4 C9 H^a H^b

(c.2) Second Choice: Atoms with the second lowest GC-GPRI values at each $\Delta\mu$ and κ.

$\Delta\mu/\kappa$	1	0.8	0.6	0.4	0.2	0	-0.2	-0.4	-0.6	-0.8	-1
0	-0.136	-0.123	-0.109	-0.095	-0.082	-0.068	-0.055	-0.041	-0.027	-0.014	
0.2	-0.136	-0.131	-0.125	-0.119	-0.117	-0.112	-0.107	-0.102	-0.116	-0.132	-0.147
0.4	-0.136	-0.139	-0.141	-0.148	-0.152	-0.155	-0.174	-0.204	-0.234	-0.264	-0.294
0.6	-0.136	-0.147	-0.162	-0.174	-0.186	-0.217	-0.262	-0.307	-0.352	-0.397	-0.442
0.8	-0.136	-0.155	-0.179	-0.200	-0.231	-0.291	-0.351	-0.410	-0.470	-0.529	-0.589
1	-0.136	-0.167	-0.196	-0.226	-0.290	-0.365	-0.439	-0.513	-0.588	-0.662	-0.736

C1 C4 C9 H^a H^b

(d.1) First Choice: Atoms with the lowest GC-GPRI values at each $\Delta\mu$ and κ.

$\Delta\mu/\kappa$	1	0.8	0.6	0.4	0.2	0	-0.2	-0.4	-0.6	-0.8	-1
0	-0.129	-0.116	-0.103	-0.090	-0.078	-0.065	-0.052	-0.039	-0.026	-0.013	
0.2	-0.129	-0.125	-0.120	-0.116	-0.112	-0.121	-0.132	-0.144	-0.155	-0.167	-0.178
0.4	-0.129	-0.133	-0.137	-0.151	-0.181	-0.210	-0.239	-0.269	-0.298	-0.327	-0.357
0.6	-0.129	-0.142	-0.158	-0.205	-0.252	-0.299	-0.346	-0.393	-0.441	-0.488	-0.535
0.8	-0.129	-0.150	-0.193	-0.258	-0.323	-0.388	-0.453	-0.518	-0.583	-0.648	-0.713
1	-0.129	-0.159	-0.229	-0.312	-0.395	-0.477	-0.560	-0.643	-0.726	-0.809	-0.891

C1 C4 C9 H^a H^b

(d.2) Second Choice: Atoms with the second lowest GC-GPRI values at each $\Delta\mu$ and κ.

$\Delta\mu/\kappa$	1	0.8	0.6	0.4	0.2	0	-0.2	-0.4	-0.6	-0.8	-1
0	-0.129	-0.116	-0.103	-0.090	-0.077	-0.064	-0.052	-0.039	-0.026	-0.013	
0.2	-0.129	-0.124	-0.120	-0.116	-0.111	-0.107	-0.103	-0.099	-0.115	-0.131	-0.148
0.4	-0.129	-0.133	-0.137	-0.142	-0.146	-0.150	-0.171	-0.202	-0.233	-0.264	-0.295
0.6	-0.129	-0.141	-0.155	-0.167	-0.180	-0.214	-0.260	-0.306	-0.352	-0.397	-0.443
0.8	-0.129	-0.150	-0.172	-0.193	-0.228	-0.288	-0.349	-0.409	-0.470	-0.530	-0.591
1	-0.129	-0.158	-0.189	-0.219	-0.287	-0.362	-0.437	-0.513	-0.588	-0.663	-0.739

C1 C4 C9 H^a H^b

(e.1) First Choice: Atoms with the lowest GC-GPRI values at each $\Delta\mu$ and κ.

$\Delta\mu/\kappa$	1	0.8	0.6	0.4	0.2	0	-0.2	-0.4	-0.6	-0.8	-1
0	-0.125	-0.112	-0.100	-0.087	-0.075	-0.062	-0.050	-0.037	-0.025	-0.012	
0.2	-0.125	-0.121	-0.117	-0.113	-0.120	-0.130	-0.140	-0.151	-0.161	-0.171	-0.181
0.4	-0.125	-0.129	-0.136	-0.164	-0.193	-0.221	-0.249	-0.277	-0.306	-0.334	-0.362
0.6	-0.125	-0.138	-0.172	-0.219	-0.265	-0.311	-0.358	-0.404	-0.451	-0.497	-0.543
0.8	-0.125	-0.147	-0.208	-0.273	-0.337	-0.402	-0.466	-0.531	-0.595	-0.660	-0.724
1	-0.125	-0.162	-0.245	-0.327	-0.410	-0.492	-0.575	-0.658	-0.740	-0.823	-0.905

C1 C4 C9 H^a H^b

(e.2) Second Choice: Atoms with the second lowest GC-GPRI values at each $\Delta\mu$ and κ.

$\Delta\mu/\kappa$	1	0.8	0.6	0.4	0.2	0	-0.2	-0.4	-0.6	-0.8	-1
0	-0.125	-0.112	-0.100	-0.087	-0.075	-0.062	-0.050	-0.037	-0.025	-0.012	
0.2	-0.125	-0.120	-0.116	-0.112	-0.109	-0.105	-0.101	-0.103	-0.117	-0.132	-0.147
0.4	-0.125	-0.128	-0.134	-0.139	-0.144	-0.148	-0.176	-0.206	-0.235	-0.264	-0.293
0.6	-0.125	-0.136	-0.151	-0.165	-0.178	-0.220	-0.264	-0.308	-0.352	-0.396	-0.440
0.8	-0.125	-0.145	-0.169	-0.190	-0.235	-0.294	-0.352	-0.411	-0.470	-0.528	-0.587
1	-0.125	-0.155	-0.186	-0.220	-0.294	-0.367	-0.440	-0.514	-0.587	-0.660	-0.733

C1 C4 C9 H^a H^b

(**f.1**) **First Choice:** Atoms with the lowest GC-GPRI values at each $\Delta\mu$ and κ.

$\Delta\mu/\kappa$	1	0.8	0.6	0.4	0.2	0	-0.2	-0.4	-0.6	-0.8	-1
0	-0.144	-0.130	-0.116	-0.101	-0.087	-0.072	-0.058	-0.043	-0.029	-0.014	
0.2	-0.144	-0.139	-0.133	-0.127	-0.121	-0.115	-0.126	-0.138	-0.151	-0.164	-0.177
0.4	-0.144	-0.147	-0.149	-0.152	-0.171	-0.201	-0.232	-0.262	-0.292	-0.323	-0.353
0.6	-0.144	-0.155	-0.166	-0.193	-0.241	-0.289	-0.337	-0.385	-0.433	-0.482	-0.530
0.8	-0.144	-0.164	-0.183	-0.246	-0.312	-0.378	-0.443	-0.509	-0.575	-0.640	-0.706
1	-0.144	-0.172	-0.216	-0.299	-0.383	-0.466	-0.549	-0.633	-0.716	-0.799	-0.883

C1	C4	C9	H^a	H^b

(**f.2**) **Second Choice:** Atoms with the second lowest GC-GPRI values at each $\Delta\mu$ and κ.

$\Delta\mu/\kappa$	1	0.8	0.6	0.4	0.2	0	-0.2	-0.4	-0.6	-0.8	-1
0	-0.144	-0.130	-0.115	-0.101	-0.087	-0.072	-0.058	-0.043	-0.029	-0.014	
0.2	-0.144	-0.138	-0.132	-0.126	-0.119	-0.113	-0.109	-0.103	-0.111	-0.128	-0.146
0.4	-0.144	-0.146	-0.148	-0.150	-0.154	-0.157	-0.163	-0.195	-0.227	-0.259	-0.291
0.6	-0.144	-0.154	-0.165	-0.177	-0.188	-0.204	-0.250	-0.297	-0.344	-0.390	-0.437
0.8	-0.144	-0.163	-0.181	-0.203	-0.222	-0.277	-0.338	-0.399	-0.460	-0.521	-0.583
1	-0.144	-0.171	-0.200	-0.228	-0.274	-0.349	-0.425	-0.501	-0.577	-0.653	-0.728

C1	C4	C9	H^a	H^b
